# Controlled Growth of Large SiO_2_ Shells onto Semiconductor Colloidal Nanocrystals: A Pathway Toward Photonic Integration

**DOI:** 10.1021/acsanm.3c05223

**Published:** 2024-02-12

**Authors:** Sergio Fiorito, Matteo Silvestri, Matilde Cirignano, Andrea Marini, Francesco Di Stasio

**Affiliations:** † Photonic Nanomaterials, Istituto Italiano di Tecnologia, 16163 Genoa, Italy; ‡ Dipartimento di Scienze Fisiche e Chimiche, Università degli studi dell’Aquila, 67100 L’Aquila, Italy; § Dipartimento di Chimica e Chimica Industriale, Università degli Studi di Genova, 16146 Genoa, Italy

**Keywords:** SiO_2_, photonics, design of experiments, quantum dots, colloidal nanocrystals, CdSe/CdS

## Abstract

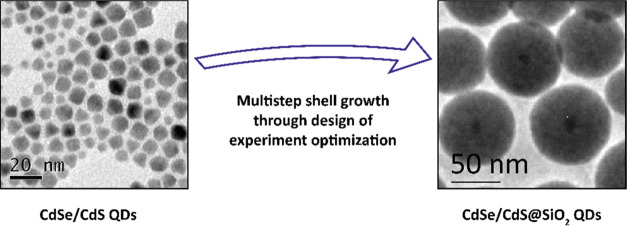

The growth of SiO_2_ shells on semiconductor nanocrystals is an established procedure and it is widely employed to provide dispersibility in polar solvents, and increased stability or biocompatibility. However, to exploit this shell to integrate photonic components on semiconductor nanocrystals, the growth procedure must be finely tunable and able to reach large particle sizes (around 100 nm or above). Here, we demonstrate that these goals are achievable through a design of experiment approach. Indeed, the use of a sequential full-factorial design allows us to carefully tune the growth of SiO_2_ shells to large values while maintaining a reduced size dispersion. Moreover, we show that the growth of a dielectric shell alone can be beneficial in terms of emission efficiency for the nanocrystal. We also demonstrate that, according to our modeling, the subsequent growth of two shells with increasing refractive index leads to an improved emission efficiency already at a reduced SiO_2_ sphere radius.

## Introduction

Colloidal semiconductor nanocrystals (NCs) are a mature technology that finds application in consumer electronic products such as displays.^1^ Most of optoelectronic applications of NCs exploit them in an ensemble (e.g., as films or dispersed in a matrix) and make use of their high photoluminescence efficiency, color tunability, and color purity. In fact, such properties have been investigated and developed thoroughly in the last 3 decades for a variety of different compositions.^2^ Nonetheless, NCs present exploitable properties at the nanoscale too; for example, a single NC acts as a single-photon emitter^3^ or it can be used for other localized phenomena.^4^ Similar to large-size optoelectronic devices (>1 mm), at the nanoscale, light out-coupling, and more generally light-control, is of paramount importance. In literature, one can find a variety of examples where single NCs have been coupled with photonic nanostructures such as antennas,^5,6^ microcavities,^7^ and waveguides.^8,9^ In most of these cases, the photonic component was prepared via microfabrication tools on a substrate where the emitting NCs were deposited either previously or after the fabrication step. A novel approach would be to combine photonic components directly onto the colloid surface, thus obtaining a single material presenting efficient and tunable photoluminescence together with a controlled emission (e.g., control over directionality, polarization, etc.). However, a major issue preventing such integration is the different dimensions of the two components; in fact, NCs are typically less than 20 nm in size,^2^ while photonic components are much larger (>100 nm). To envision a future colloidal integration of the two systems, NCs must be enlarged in size maintaining their characteristic emission properties. Silicon oxide shells (SiO_2_) are a promising candidate to enable such approach as procedures to growth them on top of NCs are already available in literature, enabling an inert shell with relatively low impact on the optical properties of the embedded nanocrystal.^10−12^ Such SiO_2_ shells can be employed as a positive resist for the integration of photonic components via nanofabrication or as a scaffold for building multilayer structures similar to distributed Bragg reflectors.^13^ The only detrimental property of enclosing NCs into a SiO_2_ shell is the electrical insulating nature of the latter, which, in turn, prevents the electrical driving of the embedded semiconductor emitter. A notable example of light-control integration through SiO_2_ shells is the work from Ji et al., where the authors were able to fabricate a plasmon microcavity directly onto a NC and enhance the emission rate.^4^ Another example is the exploitation of SiO_2_ shells to increase the size of CdSe/CdS core–shell NCs for the fabrication of single-photon emitting arrays.^14,15^ Recently, SiO_2_^16^ or similar inorganic matrixes^17^ were also exploited for the shelling of CsPbX_3_ (X = Cl, Br, and I) NCs. Yet, in most cases, the SiO_2_ shell thickness is relatively small or presents a high size dispersion. Moreover, the different procedures exploited for SiO_2_ shell growth suffer from low reproducibility and tunability due to their complex mechanisms, and most of the chemical procedures are developed exploiting the standard one-variable-at-a-time (OVAT) approach.^18,19^ These considerations motivated us to carry out a systematic study of SiO_2_ shell growth aiming at identifying a procedure to consistently obtain larger and monodispersed nanoparticles. We made use of a full-factorial “design of experiments” (DoE) approach^20,21^ that enabled us to optimize the experimental work and assess the reproducibility of our findings. Exploiting a DoE and two well-known methods to grow the SiO_2_ shell (reverse microemulsion and Stöber), we were able to consistently grow a thick SiO_2_ shell, increasing the total diameter of NCs up to 95 nm. Importantly, large SiO_2_ shells could also enhance the emission of NCs on their own, facilitating light extraction. In fact, the dipolar emission process of an NC embedded within a dielectric medium can lead to a larger far-field intensity owing to the enhanced photon momentum. However, when such an emission process takes place in a structured environment, e.g., a SiO_2_ shell, the reflection of spherical waves from the shell boundary produces emission quenching when its radius becomes smaller than λ/2π. Therefore, engineering dielectric shell thicknesses could also be of paramount importance for far-field emission tuning.

## Experimental Section

### Materials

CdO (>99%), Se (>99%) and S powder (99%), and trioctylphosphine (90%) and trioctylphosphine oxide (99%) were purchased from STREM Chemicals, octadecylphosphonic acid (>99%) from PCI Synthesis, and oleic acid (90%), octadecene (90%), chloroform (>99%), methanol (>99%), isopropanol (>99%), IGEPAL CO-520, IGEPAL CO-630, IGEPAL CA630, IGEPAL CA 720, TEOS (99%), and ammonia–water (25%) from Sigma-Aldrich.

### Synthesis of CdSe/CdS Giant Shell Nanoparticles

CdSe seeds were synthesized according to the procedure described by Carbone et al.,^22^ exploiting a temperature of 380 °C. After their synthesis, the NCs were purified by precipitation with methanol, followed by centrifugation and resuspension in toluene (3 times). The as-synthesized CdSe cores had a diameter of 3.43 nm, and their concentration was determined from the absorption in chloroform, using a known sizing curve and the molar extinction coefficient at 350 nm.^23^ In order to accomplish the CdS shell growth, Cd and S-precursors were prepared separately as a 0.5 M solution of TOP-S and Cd-oleate dissolved in ODE. Next, 2.7 × 10^–8^ mol of CdSe cores was added to 3 mL of ODE and heated up to 300 °C. Afterward, 0.57 mL of both precursors’ solutions were mixed, loaded in a syringe, and added dropwise over 30 min. Finally, the samples were purified by precipitation with isopropyl alcohol, followed by centrifugation and resuspension in toluene. A second purification step was performed with methanol as an antisolvent. Samples were then suspended in cyclohexane.

### Starting Particle Concentration Measurements

First, the elemental composition of the starting CdSe/CdS samples was evaluated through elemental analyses. The measurements were performed using an inductively coupled plasma optical emission spectroscopy (ICP-OES) instrument (Thermo Fisher, iCap 6000). Twenty five microliter of particle suspension was digested in 1 mL of aqua regia in a volumetric flask overnight at room temperature. The flask was filled up with Milli-Q water, and the solution was filtered through a 0.2 μm PTFE membrane prior to the measurement. Elemental composition was used together with the known crystalline structure and size of the nanoparticles in order to calculate their molar concentration.

### Formation of Silica Shell on CdSe/CdS NCs with Single Injection

For the growth of the first silica shell on CdSe/CdS NCs, a previously reported procedure was used and optimized. Different amounts (100–816 μL) of a surfactant were dispersed in cyclohexane inside an 8 mL glass vial and stirred at room temperature for 15 min. To the same solution were added 300 μL of CdSe/CdS NCs (molar concentration = 9.6 × 10^–7^), and then, after stirring for another 15 min, different amounts of TEOS (4–76 μL) were added. The silica shell growth was accomplished by further stirring for 15 min, adding ammonia–water (25%, 15–125 μL), and overnight reaction. Reactions were then quenched, and samples were purified by three subsequent additions of ethanol and centrifugations (10, 20, and 40 min, 5000 RCF)

### Growth of Additional Silica Shells through the Stöber Approach

For the growth of additional silica layers on silica-shelled CdSe/CdS NPs through the Stöber procedure, samples were washed following the standard procedure and then transferred to 2 mL of ethanol. 75 μL of NH_3(aq)_ was added, and the dispersion was stirred for 15 min. Finally, 76 μL of TEOS was added, and the reaction batch was stirred overnight. Samples were finally purified by three subsequent additions of ethanol and centrifugations (5 min, 5000 RCF).

### Growth of Additional Silica Shells through a Reverse Microemulsion Approach

For the growth of additional silica layers on silica-shelled CdSe/CdS NPs through a reverse microemulsion procedure, samples were kept in the reaction batch of the previous shell growth without reaction quenching. To the reactant solution were added surfactant (72–216 μL), TEOS (26–76 μL), and ammonia–water (25–75 μL) each of them after 15 min stirring. The reaction is then carried out overnight, quenched, and washed with the standard procedure.

### Electron Microscopy Analyses

All of the samples were prepared for TEM and SEM analyses by drying a drop of the diluted particle suspension on 200 mesh ultrathin carbon-coated TEM copper grids. The shape and size of the bare and silica-coated samples were analyzed by conventional TEM in bright-field mode. All of the images were acquired using a JEOL JEM-1400Plus microscope operating at 120 kV. The size distribution of the nanoparticles was evaluated through the measurement of at least 200 nanocrystals. Coalescence and surface morphology of the silica-coated nanoparticles after multiple injections were studied through scanning electron microscopy using a Zeiss Gemini SEM 560, operating at 1 kV and equipped with a field-emission gun and an In-Lens detector for secondary electron detection.

### Optical Characterization

The absorption spectra were recorded with a Varian Cary 300 UV–vis–NIR spectrophotometer. Both the cyclohexane stable (bare CdSe/CdS) and ethanol stable (silica-coated CdSe/CdS) samples were prepared in a 1 cm path length quartz cuvettes. The steady-state PL measurements were performed on a Varian Cary Eclipse spectrophotometer by exciting the sample at 400 nm. The PLQY measurements were carried out using an Edinburgh FLS900 fluorescence spectrometer equipped with an integrating sphere, exciting at 400 nm using the output of a continuous xenon lamp. All NC solutions used for PLQY measurements were diluted to an optical density of around 0.1 at 400 nm.

### DLS and ζ-Potential Measurements

The hydrodynamic diameter and ζ-potential of the NCs water suspensions were determined on a Malvern Zetasizer (Nano Series, Nano ZS) instrument. Dynamic light scattering measurements were performed with a backscattered geometry and employing a 632 nm laser source. For each sample, three independent measurements were taken, and each was the average of 12 acquisitions.

## Results and Discussion

**Figure 1:**
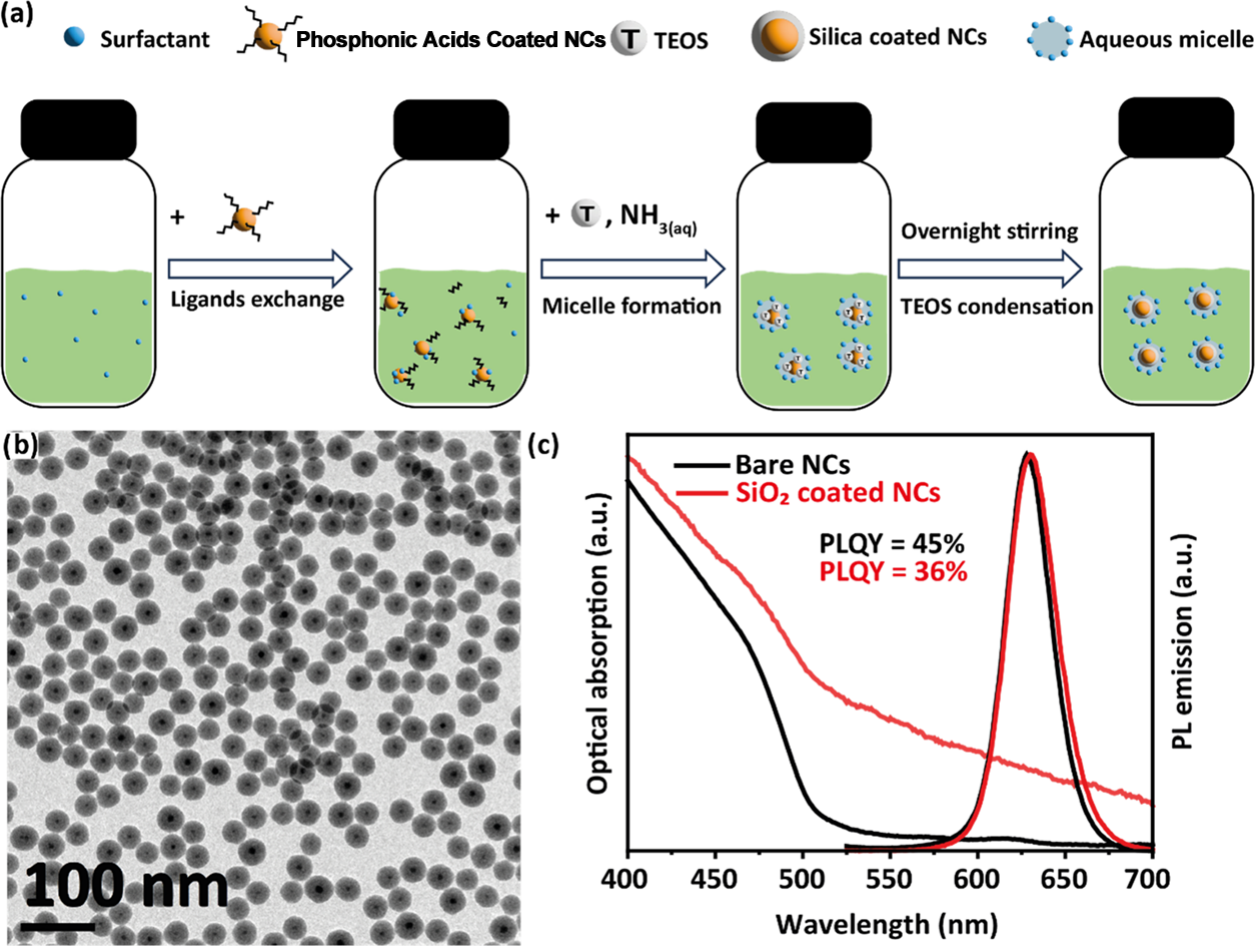
(a) Schematic of the SiO_2_ growth procedure. (b) Representative TEM bright field image of 34 ± 2 nm SiO_2_-coated CdSe/CdS NCs. (c) Normalized optical absorption and PL emission curves for CdSe/CdS NCs before (black curves) and after (red curves) SiO_2_ coating.

Our study started with the growth of a first SiO_2_ shell on the surface of presynthesized^24^ CdSe/CdS NCs (7 nm average diameter, see Figure S1) through a water-in-oil procedure called reverse microemulsion growth (Figure 1a). Such procedure can be applied to many different hydrophobic NCs (semiconductors, metal oxides, metals, and upconverting nanoparticles).^25−28^ The growth starts with an amphiphilic polymer (or surfactant) solution in cyclohexane. When phosphonic acid-coated CdSe/CdS NCs are injected into this solution, a ligand exchange takes place between the polymer and the native ligands of the NCs. The addition of tetraethyl orthosilicate (TEOS) and ammonia–water to the NC dispersion triggers the formation of aqueous micelles, inside which both NCs and TEOS enter. Overnight stirring leads to TEOS hydrolysis and condensation inside the micelles, which act as microreactors, thus controlling the shell thickness and growth rate. The standard procedure leads to spherical SiO_2_-coated NCs with an average diameter of 34 ± 2 nm, corresponding to a shell thickness Δ*r*_s_ of 13.5 nm, defined as Δ*r*_s_ = <*r*_s_>_tot_ – <*r*_s_>_NC_, where <*r*_s_>_tot_ and <*r*_s_>_NC_ are the average spherical radii of SiO_2_-coated NCs and the bare NCs, respectively, as determined via TEM analysis (Figure 1b). Notably, even if the size dispersion of the starting CdSe/CdS NC sample is quite high (≈25%), the monodispersity of the sample after the growth of the SiO_2_ shell is strongly reduced (<10%). Most importantly, on an average sample, 97% of SiO_2_ shells contain one particle, while only 3% of them are empty. In all our optimized experiments, no or very few groups of NCs (number of NCs > 1) covered by the same SiO_2_ shell were found.

Photoluminescence (PL) of the SiO_2_-covered nanoparticles (Figure 1c) does not significantly differ from that of the starting NCs, while their absorption only differs for an increased scattering contribution. However, the photoluminescence quantum yield (PLQY) of the samples drops from 45 ± 5%, in the bare sample, to 36 ± 4% for CdSe/CdS/SiO_2_ NCs. This decrease in PLQY has already been reported in literature, and it is typically assigned to emission quenching caused by the ligand exchange happening during the first step of the SiO_2_-coating process. Indeed, the emission quenching can be related to the presence of hydrolyzed TEOS that can act either as an electron or hole acceptor or as a source of charged species, thus generating an electric field responsible for the PLQY decrease, as indicated in the seminal work of Koole et al.^18^ Yet, as we will discuss later, it is possible that the impact of the SiO_2_ shell on the light-extraction efficiency has been underestimated, and it might be also responsible for the drop in PLQY.

To be able to effectively integrate photonic structures with sizes comparable to the wavelength of visible light, the SiO_2_-coating procedure must be precisely optimized. In particular, the possibility of accurately tuning the thickness of the shell up to large values while maintaining a high monodispersity and low aggregation is of key importance for the quality of the resulting sample. For this reason, we investigated and optimized the influence of all parameters on the size and quality of the grown shell. The growth of the SiO_2_ shell during the time was monitored measuring its size at different time steps: after the first 16 h (Figure S2a, corresponding to the overnight reaction time expected for the standard procedure) and then after 1, 5, and 6 more days (Figure S2b–d). The obtained sizes (Figure S2e) indicated no further thickness increase for the extended reaction time. Few papers report an important influence of the hydrophilic chain length of the surfactant on shell thickness.^29,30^ Therefore, we performed experiments employing other than the standard and widely used polyoxyethylene(5) nonylphenylether (Figure S3a), also similar polymers with different average chain lengths (with 9 and 12 oxyethylene units). The experiments exploiting the polymer with the longer hydrophilic chain (12 oxyethylene units, it was reported to give the best results in terms of SiO_2_ shell thickness) in our system gave particles of very low quality with a large amount of empty SiO_2_ (see Figure S3b). This seems to be related to the alkyl chain of the polymer used; indeed, the only molecule with an average of 12 units of oxyethylene that was found on the market has an isooctylphenyl hydrophobic termination (upper insert of Figure S3b) instead of the linear nonylphenyl moiety commonly found in 5 or 9 oxyethylene units’ polymer. If one compares the results obtained with polyoxyethylene(9) nonylphenylether (Figure S3c) with polyoxyethylene(9) isooctylphenylether (Figure S3d), the presence of the ramified alkyl chain leads to a higher amount of free SiO_2_ and to a low sample quality (i.e., aggregation, lack of shape and size control). In general, our results show that the best compromise in terms of SiO_2_ shell thickness and particle quality is found when employing polyoxyethylene(9) nonylphenylether (total particle average size of 38 ± 3 nm, corresponding to Δ*r*_s_ = 15.5 nm, Figure S3c), and consequently, we carried out all further experiments employing this polymer as a surfactant. The mechanism of the SiO_2_ shell growth on hydrophobic nanocrystals is complex, and the results obtained are governed by multiple parameters, each of them potentially depending on the others.^18,31^ Only recently, Harman et al.^32^ studied the silica coating of iron oxide nanoparticles through reverse microemulsion exploiting a design of experiments (DoE)^20^ approach, investigating the influence of TEOS and NH_4_OH concentration and of the number of TEOS fractionated addition on the resulting samples’ quality. To the best of our knowledge, this has never been done for the reverse microemulsion procedure applied to CdSe/CdS nanocrystals. For this reason, instead of optimizing the shell growth using the standard OVAT (one-variable-at-a-time) approach, often employed in colloidal nanoparticle synthesis, we made use of a DoE approach.^20^ In particular, here we exploit a two-level full-factorial design,^21^ a type of DoE that is particularly useful for this kind of experiments, given that (i) it requires a relatively small number of experiments, (ii) the results obtained from the experiments can be shown and interpreted graphically or by means of simple arithmetic, and (iii) it can be extended to unexplored areas of the experimental domain through the so-called sequential assembly. In a full-factorial design (also called a 2^*k*^ design), *k* factors vary on two levels. In the case of our DoE, the seed NC concentration in the reaction environment is kept constant at 8.73 × 10^–8^ M and the factors (or variable) of interest are the polymer, TEOS, and NH_3(aq)_ concentrations (thus *k* = 3). For each of these variables, we set two levels defined as +1 for the upper one and −1 for the lower one. In particular, we wanted to start studying the behavior of our experimental system around the conditions typically used in the standard procedure. Consequently, for each parameter object of study, we moved symmetrically below (−1 level, first line in Table 1) and above (+1 level, third line in Table 1) from the standard procedure’s parameters (second line in Table 1). Most importantly, we wanted to study a large enough experimental domain in order to achieve the largest size possible. Therefore, our ranges were defined in order to have a large cubic experimental domain with the only restriction of the experimental limitations (e.g., reactant viscosity, micropipettes accuracy, etc.). In this way, we studied a large enough cubic experimental domain of which the literature-reported procedures’ parameters represent the central point.

**Table 1:** Levels and Corresponding Concentrations Employed in the First DoE

level	polymer [M]	TEOS [M]	NH_3(aq)_ [M]
–1	1.12 × 10^–1^	5.43 × 10^–3^	6.05 × 10^–2^
starting	2.675 × 10^–1^	3.2565 × 10^–2^	1.8125 × 10^–1^
+1	4.23 × 10^–1^	5.97 × 10^–2^	3.02 × 10^–1^

**Figure 2:**
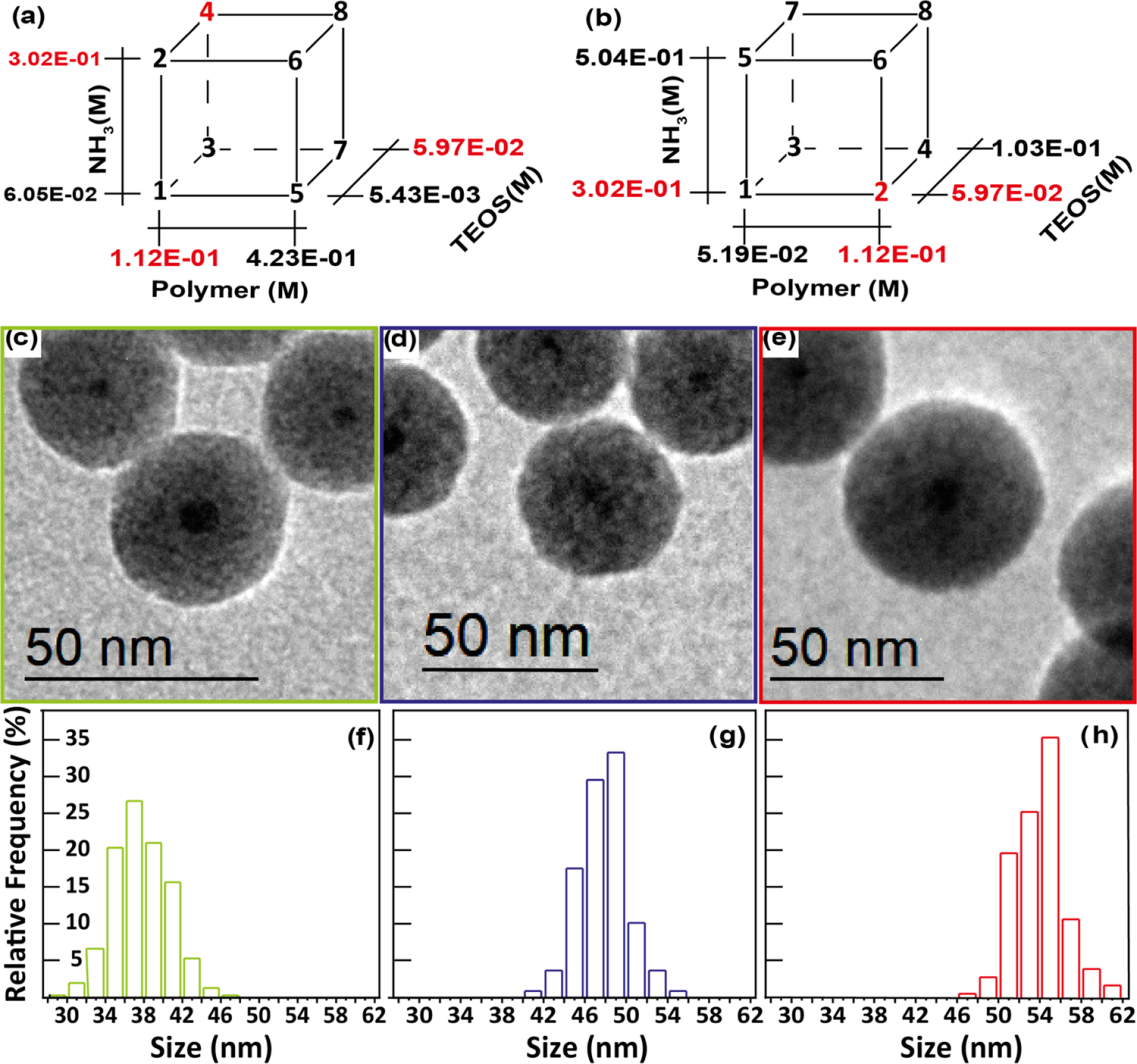
(a, b) Schematics of the two sets of DOE performed. (c–e) Representative TEM bright field image of a particles obtained with the unoptimized procedure (c) after the first DOE and (d, e) after the second DOE and (f–h) their respective size dispersion graphs.

To evaluate the results of each of the performed experiments, usually, a numerical response (or output variable) must be chosen; however, in our case, shell thickness is not sufficient alone to describe the quality of the various obtained samples. Consequently, for our experiments, also other qualitative observations such as the size dispersion, the presence of free SiO_2_ (i.e., SiO_2_ not shelling CdSe/CdS NCs) or the aggregation of the particles were considered as outputs. Accordingly, in our case, the DoE approach was employed only to study the behavior of our system inside the experimental domain while no numerical modeling was carried out. Nevertheless, the same approach can also be used in different cases to create a model of the responses and predict values outside of the experimental points. For our first DoE, we carried out a set of eight different experiments (plus replicas), each of them being one vertex of the cube representing the experimental domain object of study (see Figure S4a and Table S1). From the obtained results, it is clear how all four experiments performed with the lower concentration of TEOS only lead to aggregated (samples 1–2–5) or no (sample 6) SiO_2_-shelled NCs (see Figure S4b–f). On the contrary, among the four experiments using a larger amount of TEOS, the best results are obtained when the concentration of polymer is kept low (samples 3 and 4, Figure S4d,e) since at larger values the samples are either flower-like shaped (sample 7, Figure S4g) or contain large core-free SiO_2_ nanoparticles (sample 8, Figure S4h). Among these two selected samples, the one obtained with an increased amount of NH_3(aq)_ (sample number 4) is the one in which the thickness of the grown SiO_2_ shell is larger (total particle size of 45.5 ± 2.5 nm, Δ*r*_s_ = 19.25 nm). Usually, the simple exploitation of a DoE approach allows the optimization of experimental outputs without deepening the study of the reaction mechanism. However, in our case, some of the trends observable in the multivariate approach can be explained with the effects of the three reactants that were already evidenced exploiting OVAT studies. Indeed, apart from the case of sample 6 in which no particles at all are precipitated, increasing the concentration of NH_3(aq)_ always has the effect of increasing micelles’ and, consequently, particles’ sizes. Also, when polymer concentration is too high, the number of micelles exceeds that of particles and free SiO_2_ nanospheres are formed.^33^ Finally, TEOS being the silica source in the reaction, its shortage causes either no shell formation or collapsing of the micelles, resulting in the aggregation of the final product. Already after this first set of DoE experiments, we can increase the average thickness of the SiO_2_ shell (Δ*r*_s_) synthesized with one growth step from 15.5 to 19.25 nm. Following these first results, a new set of 8 experiments was planned. In particular, the new experimental domain shared one of the vertices with the old one (corresponding to sample *n*° 2 in the latter and the best sample n° 4 in the former). Basically, the new cubic space of experiments is placed toward higher values of TEOS and ammonia concentrations but lower amounts of polymer. The relations between the two designs and the concentration values for the new one is shown in Figure 2a,b and Table 2, respectively. In this second set of experiments (see Figure S5 and Table S2), all samples obtained with the lower amount of polymer (samples number 1–3–5–7, Figure S5b–d,f–h) had to be discarded because of the presence of a large amount of empty SiO_2_ nanoparticles (presumably due to the amount of polymer being insufficient for micelles formation). Among the four remaining samples, the two with lower amounts of ammonia (sample numbers 2 and 4, Figure S5) present the best results, with experiment number 4 resulting in the best compromise between shell thickness and particle quality. Indeed, it has larger particles (the total particle size being 52.5 ± 2.5 nm, Δ*r*_s_ = 22.75 nm, Figure S5e) and without the presence of free silica shells that is evidenced when ammonia content is increased (Figure S5g-i).

**Table 2:** Levels and Corresponding Concentrations employed in Second DoE

level	polymer [M]	TEOS [M]	NH_3(aq)_ [M]
–1	5.19 × 10^–2^	5.97 × 10^–2^	3.02 × 10^–1^
+1	1.12 × 10^–1^	1.03 × 10^–1^	5.04 × 10^–1^

Therefore, we observe a substantial increase of SiO_2_ shell thickness obtained with a single injection through a reverse microemulsion procedure: from 15.5 nm (initial standard procedure, see Figure 2c) to 19.25 nm (after the first DoE, see Figure 2d), and, finally, to 22.75 nm (with the second DoE, see Figure 2e, all size dispersions for these samples are shown in Figure 2f–h).

**Figure 3:**
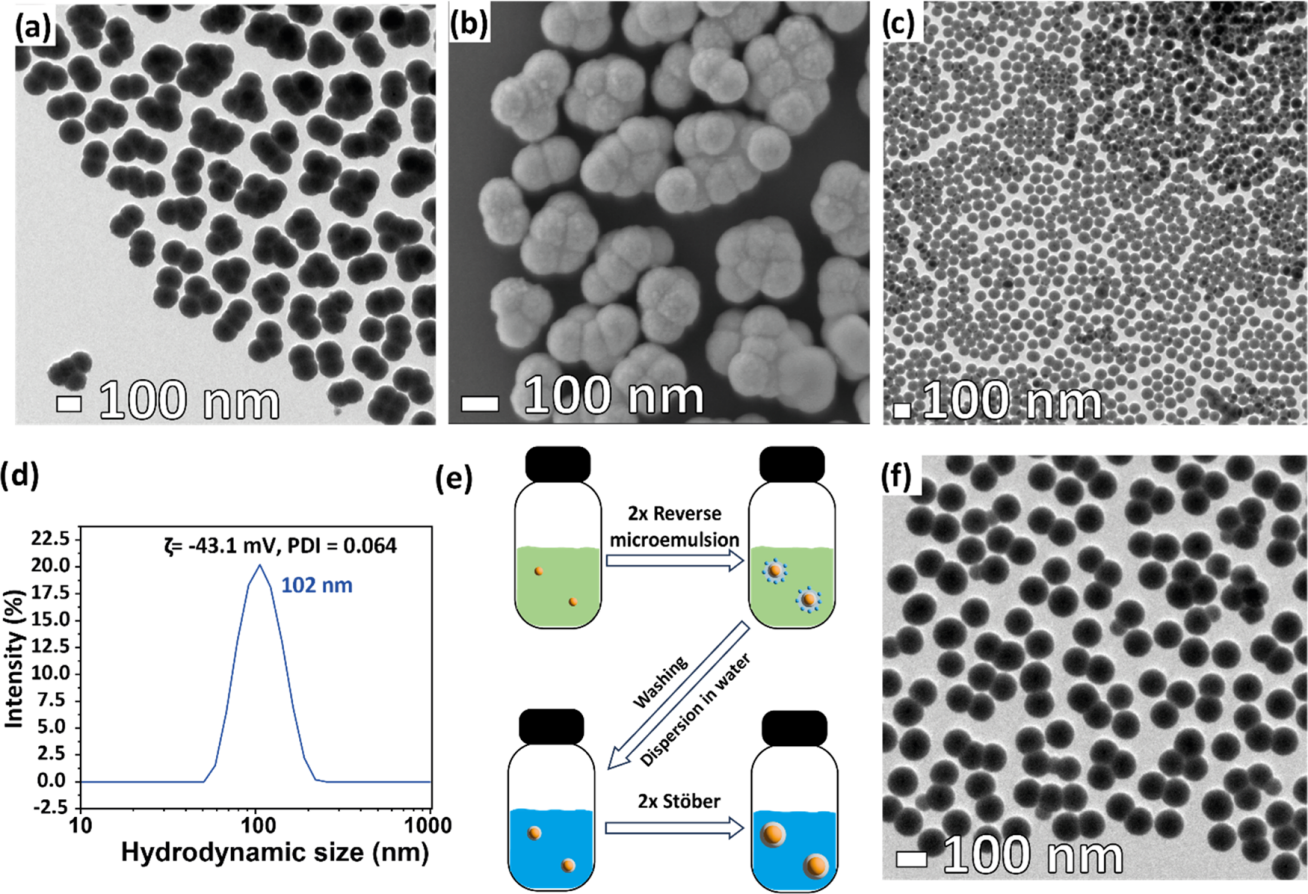
Representative TEM bright field image (a) and SEM image (b) of SiO_2_-coated samples produced employing 1 step of reverse microemulsion followed by 2 steps of the Stöber procedure, (c) representative TEM bright field image of a sample obtained through the DOE optimization of two sequential SiO_2_ growth obtained through reverse microemulsion, (d) dynamic light scattering size dispersion graph (by intensity) of the sample obtained after two optimized steps of reverse microemulsion, (e) schematic of the entire procedure employed for the growth of the largest SiO_2_ shells, and (f) representative TEM bright field image of SiO_2_-coated samples produced employing two optimized steps of reverse microemulsion followed by two steps of Stöber procedure.

As expected with the increasing thickness of the SiO_2_ shell, the scattering contribution in the absorption spectrum is increased (see Figure S6), while on the other hand, the PL emission is not significantly modified. Of particular interest are the dynamic light scattering (DLS) and ζ-potential measurements performed on the two samples (see Figure S7). Even though the optimized sample is 14.5 nm larger than the starting one, the hydrodynamic size is reduced from 116 to 92 nm, accounting for the increased colloidal stability of the sample and reduced aggregation. Accordingly, the polydispersity index (PDI) decreases from 0.256 to 0.110, due to the increased quality of the sample. Together with the hydrodynamic size and PDI, the zeta potential (ζ) of the particles is reduced from −23.2 to −35.4 mV. This behavior can be attributed to the increased size of the particles^34^ and to the reduced amount of amphiphilic surfactant on particle surfaces,^35^ thus accounting for the increased colloidal stability. An additional growth of the SiO_2_ shell can be further obtained via a multiple-injection approach.^31,33^ At first, a sample of optimized SiO_2_-coated NCs (average diameter size of 52.5 nm, PDI of 0.110 and ζ = −35.4 mV) was purified, transferred into water, and subjected to two consecutive Stöber procedures.^36^ Interestingly, the surfaces of the particles obtained through this different approach are much rougher than the previous ones (see Figure S8), accounting for the different growth speeds and mechanisms.^37^ However, as expected from previously reported findings,^35^ particles that are already aggregated after a first Stöber injection (see Figure S9) at the end of the second step are coalescing into large accumulates of two or more particles (see TEM image in Figure 3a and SEM image in Figure 3b).

Consequently, following what was reported by Kembuan et al.,^35^ a further reverse microemulsion procedure step must be added before the Stöber growth, and, once again, we set up a new DoE to find the experimental parameters able to lead to the best results. For this third DoE, the molar ratios of the three reactants were considered (Table 3). To be more specific, the +1 level of each reactant corresponds to the addition of the exact same amount used for the first growth step, while the −1 level corresponds to one-third of that amount. Among all of the samples obtained (see Figure S10 and Table S3), the best results are the ones achieved when a low amount of TEOS and a high amount of ammonia–water are added. Sample number 6 (synthesized with a larger polymer to NCs ratio, see Figure 3c) has a total size of 73 nm with a standard deviation of only 4 nm (≈5%, Δ*r*_s_ = 33 nm). The PDI value for this sample (see Figure 3d) is reduced further to 0.064 and the zeta potential below −40 mV, thus making the particles suitable for further shell growth with a Stöber procedure without inducing aggregation.^35^ Interestingly, the size dispersion is reduced to a point (≈4%) where the obtained SiO_2_-coated NCs seem to spontaneously organize themselves in a face-centered cubic lattice (Figure 3c), similar to what observed for nanospheres used for the fabrication of photonic crystals^38,39^ or colloidal lithography.^40^ Transferring to water the samples obtained through the two optimized injections and injecting more TEOS and NH_3(aq)_ following the Stöber approach results in reduced aggregation and coalescence (see Figure S11, if compared to what happened when the Stöber procedure was used after only one reverse microemulsion iteration). Ultimately, following the optimized procedure (that is, two reverse microemulsion steps followed by two subsequent Stöber growth, Figure 3e), we can obtain particles (see Figure 3f) with an average diameter of 95 ± 4 nm (Δ*r*_s_ = 44 nm), reduced aggregation, and size dispersion (4%). Importantly, the optimized process has an entire duration of 5 days (4 nights) with limited hands-on working time (about 2 h per step for a total of 8 h).

**Table 3:** Levels and Corresponding Ratios with NPs Concentrations Employed in Third DoE

level	polymer/NPs	TEOS/NPs	NH_3_/NPs
–1	1.7 × 10^6^	1.6 × 10^6^	4.6 × 10^6^
+1	2.6 × 10^6^	2.4 × 10^6^	6.9 × 10^6^

As briefly discussed above, large SiO_2_ shells can enhance the emission of NCs by facilitating light extraction. Given the relatively large size of the SiO_2_ shells that we grew, we investigated the influence of a SiO_2_ shell having different structures (glass, silica, and quartz) on the emission of a NC through a dedicated modeling.^41,42^ We performed the modeling considering glass, silica, and quartz as these three structures of SiO_2_ possess different refractive indexes (*n*_in_ = 1.3, 1.4, and 1.45, respectively),^41,42^ in an attempt to approximate the refractive index of our SiO_2_ shell. To model the NC emission process, we assumed that it is placed at the center of a dielectric sphere with the refractive index  and it has negligible physical dimensions, thus leading to purely point-like dipolar radiation. The NC emission modeling (see the Supporting Information) involves (i) the expansion of the radiated field within the dielectric sphere and the outer medium (air in our calculations) in terms of vector spherical harmonics^43^ and (ii) matching of boundary conditions (BCs) for the continuity of the displacement vector normal component, electric field tangential components, and of the entire magnetic field. This enables the calculation of the total radiated power in the far field through the integration of the dipolar Poynting vector over a sphere with radius *R* ≫ λ. To assess the out-coupling properties of the SiO_2_ shell, we define the structural radiative efficiency as the ratio between such radiated power and the one radiated by an isolated dipole in air, explicitly given by
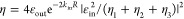
1where *R* is the sphere radius (corresponding to the previously introduced experimental variable *r*)
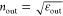
2is the external refractive index (air in our case, *n*_out_ = 1), and
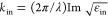
3
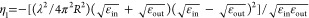
4
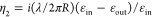
5
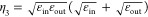
6

**Figure 4:**
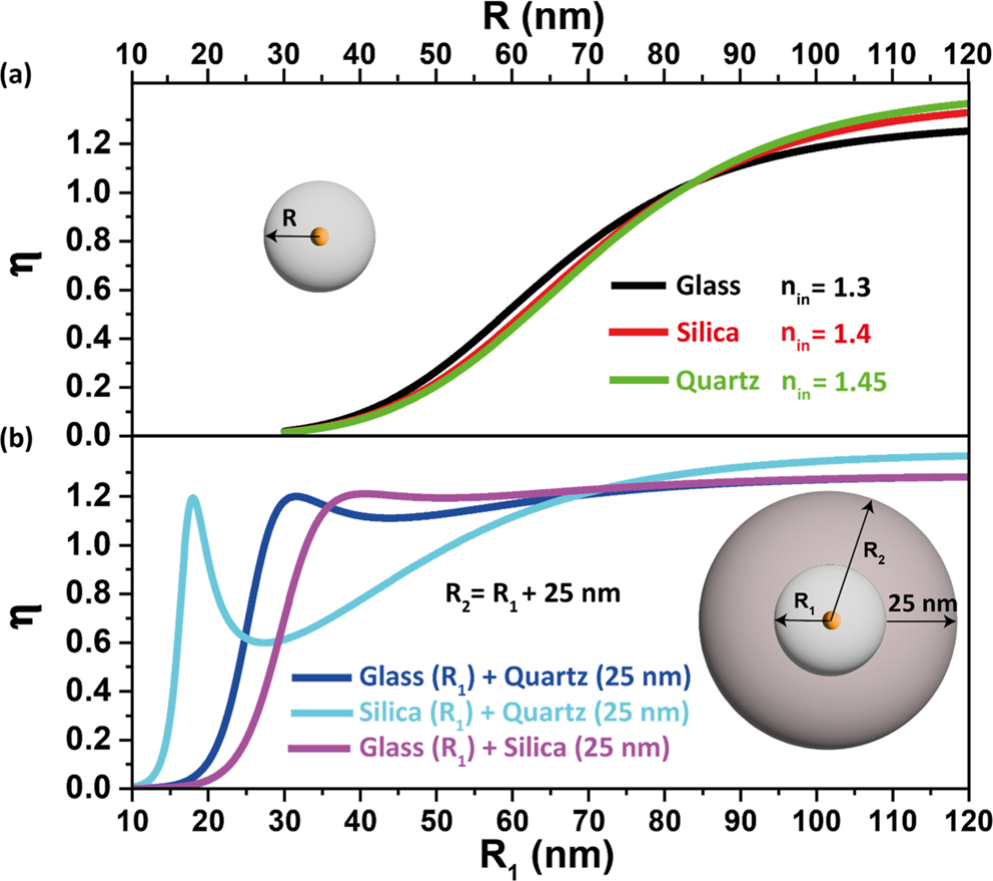
(a) Structural radiative efficiency η dependence on shell’s radius (*R*) for spherical shells composed of distinct SiO_2_ dielectric media: glass (black), silica (red), and quartz (green) with refractive indexes *n*_in_ = 1.3, *n*_in_ = 1.4, and *n*_in_ = 1.45, respectively. (b) Structural radiative efficiency η dependence on first shell’s radius (*R*1) for spherical shells composed of two distinct SiO_2_ dielectric media: glass–quartz (blue), silica–quartz (cyan), and glass–silica (magenta); the thickness of the second shell is here defined as *d* and fixed to *d* = 25 nm. The total size of the sphere is defined as *R*_2_ = *R*_1_ + *d*.

In Figure 4a, we depict the structural radiative efficiency dependence over the dielectric sphere radius, indicating monotonically growing behavior saturating to a plateau that depends on the type of SiO_2_ considered. For all of the considered materials (glass, silica, and quartz), we obtain an efficiency increase of 25–40% with respect to emission in air for *r* > 85 nm. The increase in efficiency depends on the refractive index of the SiO_2_ shell, with a more pronounced enhancement observed for quartz (*n*_in_ = 1.45) and a diminished one for glass (*n*_in_ = 1.3). Such behavior arises from the out-coupling efficiency dependence on ε_in_^3^, meaning that the efficiency is higher when the refractive index is higher. Physically, increasing the refractive index of the dielectric sphere produces an increase in the emitted radiation momentum density (i.e., the Poynting vector), thus enhancing the emitted power outside the dielectric-coated NC. It is important to underline that our modeling is valid for a point-like dipole, while the NCs we are using in our study have an average diameter of 7 nm. Additionally, we do not know the precise *n*_in_ value of the SiO_2_ shell, even if it falls in the range 1.3–1.45. Therefore, the estimated enhancement gives us only a qualitative indication of the potential impact of the SiO_2_ shell on the emission efficiency. Nevertheless, these findings suggest that a large SiO_2_ shell could be of use not only to couple photonic components to an emitting NC but also to enhance their emission through improved optical out-coupling.

On the other hand, a thin shell is detrimental for light extraction, and this can play an important role in the decrease of PQLY, which is often observed after the shelling procedure. In fact, often in the literature, the drop of PLQY upon SiO_2_ shelling is assigned to quenching of the emission due to surface processes,^10,18^ although our modeling indicates that a thin shell can also play a detrimental role due to back-reflection from the outer shell. Yet, we must consider that, to precisely assess the impact, one would need to consider the effect of the SiO_2_ shell also on the optical absorption of the NC and not only its emission.

Furthermore, we also modeled the situation in which an additional dielectric shell is grown on the surface of the previous one. Here, we consider the emission of an NC embedded within a nanoshell structure with inner/outer radius *R*_1_/*R*_2_ and refractive indexes  and , respectively. Similar to the method described above for spherical structures, we obtain the structural radiative efficiency by vector spherical harmonic expansion and imposition of BCs at both interfaces (shell 1/shell 2 and shell 2/air), obtaining
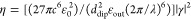
7where γ(*R*_1_,*R*_2_,ε_1_,ε_2_,ε_out_) is calculated by numerically inverting the system derived from BCs. We observe the existence of an optimal radius (Figure 4b) where out-coupling efficiency is maximized. Such optimal out-coupling efficiency is pronounced at a peculiar refractive index difference between the two-shell media and depends on the shell thickness. Such a peculiar behavior ensues from multiple reflections from outer/inner shell interfaces, which are modulated by the acquired phase of spherical waves over the shell thickness. Interestingly, our modeling seems to indicate that when a certain thickness (*d* = 25 nm) of a second shell of higher refractive index material is added, the maximum of out-coupling efficiency can be obtained at drastically lower values of the first shell radius (*R*_1_ ≈ 18 nm for the inner shell of silica with a 25 nm outer shell of quartz, cyan line in Figure 4b). As expected, we observe the disappearance of such an optimal out-coupling efficiency radius in the limit where the refractive indexes of the inner sphere and outer shell coincide. Ultimately, our theoretical calculations suggest that (i) modulating the shell thickness and composition and (ii) adding multiple subsequent shells of different materials could greatly increase light out-coupling, thus paving the way for additional studies that, exploiting the multivariate approach, will target the precise tuning of silica shell thickness and composition. In addition, these results further demonstrate the importance of achieving fine control over shell thicknesses through an experimental design such as the one here reported.

## Conclusions

Despite being an established procedure, SiO_2_ shelling onto NCs suffers from low reproducibility and difficult tunability of outputs caused by the complex reaction mechanism and its multivariate dependence on the experimental parameters. Here, by using a design of experiment approach, taking into consideration the interaction between the experimental parameters employed in the reverse microemulsion approach, we were able to increase the thickness of the first grown SiO_2_ shell from 15.5 to 22.75 nm (47% increase from the unoptimized procedure). In a similar way, also the growth of a second shell following the same procedure was optimized (45% increase from the first shell growth), and finally, through an unoptimized Stöber approach, a final shell thickness of 44 nm (33% increase from the second shell growth) has been obtained while maintaining a limited size distribution (≈4%). If needed, the multivariate optimization procedure can be exploited for further growth steps or on different procedures (i.e., Stöber procedure) to grow even larger particles. Importantly, this approach can be expanded to other types of NCs with subtle modifications. Finally, our modeling indicates that a large SiO_2_ shell could already be beneficial for the light-emission properties of NCs by enhancing optical out-coupling. Moreover, we showed that if an additional thin (*d* = 25 nm) shell of even higher refractive index material is added as an outer layer, the same efficiency increase can be obtained with a reduced thickness of the inner shell. In the future, we plan to assess the impact of SiO_2_ shells of different thicknesses and compositions on the emission properties of single NCs and study if such shells can be employed as scaffolds for further integration of photonics components such as waveguides and microcavities with colloidal emitters.

## Supporting Information Available

A Supporting Information file is available free of charge, and it contains optical modeling details, design of experiments results, and additional experimental characterization of the starting materials and of the shelled nanocrystals (PDF)
