# Supplementary material for: Controlled Growth of Large SiO2 Shells onto Semiconductor Colloidal Nanocrystals: A Pathway Toward Photonic Integration
Source: ACS Appl Nano Mater. 2024 Feb 12;7(4):3724–33. doi: 10.1021/acsanm.3c05223 (PMC10897880; doi:10.1021/acsanm.3c05223)
Supplement: Supplementary file 1 — an3c05223_si_001.pdf [file an3c05223_si_001.pdf]

Supporting Information

Controlled Growth of Large SiO_2 Shells onto Semiconductor Colloidal Nanocrystals: a Pathway Toward Photonic Integration

Sergio Fiorito[a,*], Matteo Silvestri[b], Matilde Cirignano[a,c], Andrea Marini[b] and Francesco Di Stasio[a,*]

a Photonic Nanomaterials, Istituto Italiano di Tecnologia, 16163, Genoa, Italy

b Dipartimento di Scienze Fisiche e Chimiche, Università degli studi dell'Aquila, 67100, L'Aquila, Italy

c Dipartimento di Chimica e Chimica Industriale, Università degli Studi di Genova, 16146 Genoa, Italy

Sergio Fiorito: Sergio.Fiorito@iit.it

Francesco Di Stasio: Francesco.Distasio@iit.it

Modelling:

In this supplementary material we provide some details about the theoretical model used. We consider a quantum dot placed at the center of a dielectric sphere with refractive index n. Assuming purely dipolar quantum dot emission, we model the radiative properties of the system by a radiation point-like dipole placed at the center of the dielectric sphere. The calculation involves the expansion of the electric radiated field by vector spherical harmonics.

I. Multipole expansion

In this section we briefly illustrate the multipole expansion of electric and magnetic fields. First, we remind that, for a monochromatic field with angular frequency ω, the real electric and magnetic fields are given by the Ansatz

$$\begin{cases} \mathbf{E}(\mathbf{r},t) = \mathrm{Re}\left[\mathbf{E}_0(\mathbf{r})e^{-i\omega t}\right], \\ \mathbf{H}(\mathbf{r},t) = \mathrm{Re}\left[\mathbf{H}_0(\mathbf{r})e^{-i\omega t}\right], \end{cases} \tag{1.1}$$

Maxwell's equations for such monochromatic fields provide

$$\begin{cases} \nabla\cdot\mathbf{E} = 0, \\ \nabla\cdot\mathbf{H} = 0, \\ \nabla\times\mathbf{E} = -\mu_0\partial_t\mathbf{H}, \\ \nabla\times\mathbf{H} = \varepsilon_0\varepsilon\,\partial_t\mathbf{E}, \end{cases} \quad\rightarrow\quad \begin{cases} \nabla\cdot\mathbf{E}_0 = 0, \\ \nabla\cdot\mathbf{H}_0 = 0, \\ \nabla\times\mathbf{E}_0 = i\omega\mu_0\mathbf{H}_0, \\ \nabla\times\mathbf{H}_0 = -i\omega\varepsilon_0\varepsilon\,\mathbf{E}_0, \end{cases} \tag{1.2}$$

where $\varepsilon = n^2$ is the dielectric constant of the sphere, μ_0, ε_0 are the vacuum magnetic permeability and dielectric permittivity, respectively. Then we can uncouple the electric field taking the double curl, obtaining

$$\begin{cases} \nabla^2\mathbf{H}_0 + \varepsilon\dfrac{\omega^2}{c^2}\mathbf{H}_0 = 0, \\ \mathbf{E}_0 = \dfrac{i}{\omega\varepsilon_0\varepsilon}\nabla\times\mathbf{H}_0 . \end{cases} \tag{1.3}$$

Similarly, we can eliminate the magnetic field to obtain

$$\begin{cases} \nabla^2\mathbf{E}_0 + \varepsilon\dfrac{\omega^2}{c^2}\mathbf{E}_0 = 0, \\ \mathbf{H}_0 = \dfrac{1}{i\omega\mu_0}\nabla\times\mathbf{E}_0 . \end{cases} \tag{1.4}$$

One can show that also the scalar quantities $\mathbf{r}\cdot\mathbf{E}_0$ and $\mathbf{r}\cdot\mathbf{H}_0$ satisfy the Helmoltz equation[1]

$$\begin{cases} \nabla^2\left[\mathbf{r}\cdot\mathbf{E}_0\right]=\mathbf{r}\cdot\nabla^2\mathbf{E}_0=-\varepsilon\dfrac{\omega^2}{c^2}\left[\mathbf{r}\cdot\mathbf{E}_0\right] , \\ \nabla^2\left[\mathbf{r}\cdot\mathbf{H}_0\right]=\mathbf{r}\cdot\nabla^2\mathbf{H}_0=-\varepsilon\dfrac{\omega^2}{c^2}\left[\mathbf{r}\cdot\mathbf{H}_0\right] . \end{cases} \quad (1.5)$$

In turn, we expand such scalar quantities in spherical harmonics

$$\begin{cases} \left[\mathbf{r}\cdot\mathbf{E}_0\right]=\sum\limits_{l=0}^{\infty}\sum\limits_{m=-l}^{l} f_{l,m}(r)Y_{l,m}(\theta,\phi) , \\ \left[\mathbf{r}\cdot\mathbf{H}_0\right]=\sum\limits_{l=0}^{\infty}\sum\limits_{m=-l}^{l} g_{l,m}(r)Y_{l,m}(\theta,\phi), \end{cases} \quad (1.6)$$

where $Y_{lm}(\theta,\phi)$ are spherical harmonic functions explicitly given by

$$Y_{l,m}(\theta,\phi)=\sqrt{\frac{(2l+1)}{4\pi}\frac{(l-m)!}{(l+m)!}}P_l^m(\cos\theta)e^{im\phi} , \quad (1.7)$$

and $P_l^m(\cos\theta)$ are the associated Legendre polynomials that can be expressed by Rodrigues' formula[2,3] as

$$P_l^m(x)=\frac{(-1)^m}{2^l l!}\left(1-x^2\right)^{m/2}\frac{d^{l+m}}{dx^{l+m}}\left(x^2-1\right)^l , \quad (1.8)$$

with the orthogonality rule[2,3]

$$\int_{-1}^{1} P_{l'}^m(x)P_l^m(x)dx=\frac{2}{(2l+1)}\frac{(l+m)!}{(l-m)!}\delta_{l,l'} . \quad (1.9)$$

In turn, the radius-dependent scalar functions $f_{l,m}(r)$ and $g_{l,m}(r)$ satisfy the equations

$$\begin{cases} \dfrac{d}{dx}\left[x^2\dfrac{df_{l,m}(x)}{dx}\right]+\left[x^2-l(l+1)\right]f_{l,m}(x)=0 , \\ \dfrac{d}{dx}\left[x^2\dfrac{dg_{l,m}(x)}{dx}\right]+\left[x^2-l(l+1)\right]g_{l,m}(x)=0 , \end{cases} \quad (1.10)$$

where $x=\dfrac{\omega}{c}r\sqrt{\varepsilon}$. We note that these equations coincide with the spherical Bessel equation for $f_{l,m}(r)$ and $g_{l,m}(r)$. In turn, solutions are given by

$$\begin{cases} f_{l,m}(x)=\alpha_{l,m}^{(1)}h_l^{(1)}(x)+\alpha_{l,m}^{(2)}h_l^{(2)}(x) , \\ g_{l,m}(x)=\beta_{l,m}^{(1)}h_l^{(1)}(x)+\beta_{l,m}^{(2)}h_l^{(2)}(x), \end{cases} \quad (1.11)$$

where $h_l^{(1)}(x)$ and $h_l^{(2)}(x)$ are spherical Hankel functions of first and second kind that can be expressed in term of the spherical Bessel function $\left(j_l(x), y_l(x)\right)$ as

$$\begin{cases} h_l^{(1)}(x) = j_l(x) + iy_l(x) = (-i)^{n+1} \dfrac{e^{ix}}{x} \sum_{m=0}^{n} \dfrac{i^m}{m!(2x)^m} \dfrac{(n+m)!}{(n-m)!}, \\ h_l^{(2)}(x) = j_l(x) - iy_l(x) = (i)^{n+1} \dfrac{e^{-ix}}{x} \sum_{m=0}^{n} \dfrac{(-i)^m}{m!(2x)^m} \dfrac{(n+m)!}{(n-m)!}, \end{cases} \tag{1.12}$$

$\alpha_{l,m}^{(1)}, \alpha_{l,m}^{(2)}, \beta_{l,m}^{(1)}$ and $\beta_{l,m}^{(2)}$ are coefficients to be found by imposing the boundary conditions (BCs) for the continuity of tangent electric field (azimuthal and zenithal components), the radial component of the displacement vector and the full magnetic field at the dielectric sphere surface $r = R$. Using Eqs. (1.3,1.4), one can define an electric multipole field of order (l,m) by

$$\begin{cases} [\mathbf{r} \cdot \mathbf{E}_0] = \sum_{l=0}^{\infty} \sum_{m=-l}^{l} \dfrac{c}{\omega\sqrt{\varepsilon}} l(l+1) f_{l,m}(r) Y_{l,m}(\theta,\phi), \\ [\mathbf{r} \cdot \mathbf{H}_0] = 0 . \end{cases} \tag{1.13}$$

Instead, a magnetic multipole field of order (l,m) is defined by

$$\begin{cases} [\mathbf{r} \cdot \mathbf{E}_0] = 0 , \\ [\mathbf{r} \cdot \mathbf{H}_0] = \sum_{l=0}^{\infty} \sum_{m=-l}^{l} \dfrac{c}{\omega\sqrt{\varepsilon}} l(l+1) g_{l,m}(r) Y_{l,m}(\theta,\phi) . \end{cases} \tag{1.14}$$

Finally, from the curl equations (1.3, 1.4) one can relate $\mathbf{r} \cdot \mathbf{E}_0$ and $\mathbf{r} \cdot \mathbf{H}_0$ to the magnetic and electric field respectively by a differential operator **L** obtaining[1]

$$\begin{cases} \mathbf{r} \cdot \mathbf{E}_0 = \dfrac{i}{\omega\varepsilon_0\varepsilon} \mathbf{r} \cdot \nabla \times \mathbf{H}_0 = \dfrac{i}{\omega\varepsilon_0\varepsilon} (\mathbf{r} \times \nabla) \cdot \mathbf{H}_0 = -\dfrac{1}{\omega\varepsilon_0\varepsilon} \mathbf{L} \cdot \mathbf{H}_0 , \\ \mathbf{r} \cdot \mathbf{H}_0 = \dfrac{1}{i\omega\mu_0} \mathbf{r} \cdot \nabla \times \mathbf{E}_0 = \dfrac{1}{i\omega\mu_0} (\mathbf{r} \times \nabla) \cdot \mathbf{E}_0 = \dfrac{1}{\omega\mu_0} \mathbf{L} \cdot \mathbf{E}_0 , \end{cases} \tag{1.15}$$

where

$$\mathbf{L} = \frac{1}{i} \mathbf{r} \times \nabla . \tag{1.16}$$

In turn, using Eqs. (1.1,1.2) we obtain electric-like transverse magnetic (TM) fields

$$
\begin{cases}
\mathbf{L}\cdot\mathbf{H}_0 = -\varepsilon_0 c\sqrt{\varepsilon}\sum_{l=0}^{\infty}\sum_{m=-l}^{l} l\left(l+1\right) f_{l,m}\left(r\right)Y_{l,m}\left(\theta,\phi\right), \\
\left[\mathbf{r}\cdot\mathbf{H}_0\right]=0, \\
\qquad\qquad\downarrow \\
\mathbf{H}_0 = -\varepsilon_0 c\sqrt{\varepsilon}\sum_{l=0}^{\infty}\sum_{m=-l}^{l} f_{l,m}\left(r\right)\mathbf{L}Y_{l,m}\left(\theta,\phi\right), \\
\mathbf{E}_0 = -\dfrac{ic}{\omega\sqrt{\varepsilon}}\sum_{l=0}^{\infty}\sum_{m=-l}^{l}\nabla\times\left[f_{l,m}\left(r\right)\mathbf{L}Y_{l,m}\left(\theta,\phi\right)\right],
\end{cases}
\tag{1.17}
$$

and magnetic-like transverse electric (TE) fields

$$
\begin{cases}
\left[\mathbf{r}\cdot\mathbf{E}_0\right]=0, \\
\mathbf{L}\cdot\mathbf{E}_0 = \dfrac{\mu_0 c}{\sqrt{\varepsilon}}\sum_{l=0}^{\infty}\sum_{m=-l}^{l} l\left(l+1\right) g_{l,m}\left(r\right)Y_{l,m}\left(\theta,\phi\right), \\
\qquad\qquad\downarrow \\
\mathbf{E}_0 = \sum_{l=0}^{\infty}\sum_{m=-l}^{l} g_{l,m}\left(r\right)\dfrac{\mu_0 c}{\sqrt{\varepsilon}}\mathbf{L}Y_{l,m}\left(\theta,\phi\right), \\
\mathbf{H}_0 = -\dfrac{ic}{\omega\sqrt{\varepsilon}}\sum_{l=0}^{\infty}\sum_{m=-l}^{l}\nabla\times\left[g_{l,m}\left(r\right)\mathbf{L}Y_{l,m}\left(\theta,\phi\right)\right].
\end{cases}
\tag{1.18}
$$

Hence, the vectorial spherical harmonics expansion of an arbitrary field with both TE and TM components is given by the superposition[3]

$$
\begin{cases}
\mathbf{E}_0 = \sum_{l=0}^{\infty}\sum_{m=-l}^{l}\left\{\dfrac{\mu_0 c}{\sqrt{\varepsilon}}\left[\beta_{l,m}^{(1)}h_l^{(1)}\left(\dfrac{\omega}{c}r\sqrt{\varepsilon}\right)+\beta_{l,m}^{(2)}h_l^{(2)}\left(\dfrac{\omega}{c}r\sqrt{\varepsilon}\right)\right]\mathbf{X}_{l,m}-\dfrac{ic}{\omega\sqrt{\varepsilon}}\nabla\times\left[\left[\alpha_{l,m}^{(1)}h_l^{(1)}\left(\dfrac{\omega}{c}r\sqrt{\varepsilon}\right)+\alpha_{l,m}^{(2)}h_l^{(2)}\left(\dfrac{\omega}{c}r\sqrt{\varepsilon}\right)\right]\mathbf{X}_{l,m}\right]\right\}, \\
\mathbf{H}_0 = \sum_{l=0}^{\infty}\sum_{m=-l}^{l}\left\{-\varepsilon_0 c\sqrt{\varepsilon}\left[\alpha_{l,m}^{(1)}h_l^{(1)}\left(\dfrac{\omega}{c}r\sqrt{\varepsilon}\right)+\alpha_{l,m}^{(2)}h_l^{(2)}\left(\dfrac{\omega}{c}r\sqrt{\varepsilon}\right)\right]\mathbf{X}_{l,m}-\dfrac{ic}{\omega\sqrt{\varepsilon}}\nabla\times\left[\left[\beta_{l,m}^{(1)}h_l^{(1)}\left(\dfrac{\omega}{c}r\sqrt{\varepsilon}\right)+\beta_{l,m}^{(2)}h_l^{(2)}\left(\dfrac{\omega}{c}r\sqrt{\varepsilon}\right)\right]\mathbf{X}_{l,m}\right]\right\},
\end{cases}
\tag{1.19}
$$

where $\mathbf{X}_{l,m}\left(r,\theta,\phi\right)$ in terms of the vector spherical harmonic $\left(\mathbf{L}Y_{l,m}\left(\theta,\phi\right)\right)$ is defined as

$$
\mathbf{X}_{l,m}\left(r,\theta,\phi\right)=\frac{1}{\sqrt{l\left(l+1\right)}}\mathbf{L}Y_{l,m}\left(\theta,\phi\right), \tag{1.20}
$$

with orthogonality properties

$$
\begin{cases}
\int_{\Omega}\mathbf{X}_{l'.m'}{}^{*}\cdot\mathbf{X}_{l.m}\,d\Omega=\delta_{l,l'}\delta_{m,m'}, \\
\int_{\Omega}\mathbf{X}_{l'.m'}{}^{*}\cdot\left(\mathbf{r}\times\mathbf{X}_{l.m}\right)d\Omega=0.
\end{cases}
\tag{1.21}
$$

II. Electric dipole in a sphere

In this section we show how to find the coefficients of equation (1.19) in the case where we place a dipole in the center of a dielectric sphere of radius R. We then consider an oscillating electric dipole $\mathbf{p}_{\text{dip}} = \mathrm{Re}\left[\mathbf{d}_{\text{dip}} e^{-i\omega t}\right]$ radiating monochromatic light in a sphere with relative dielectric permittivity $\varepsilon_{\text{in}} = \varepsilon$. The real electric and magnetic fields over all space are in turn given by

$$\begin{aligned} \mathbf{E}(\mathbf{r},t) &= \mathrm{Re}\left\{\left[\mathbf{E}_{<}(\mathbf{r})\theta(r_0 - r) + \mathbf{E}_{>}(\mathbf{r})\theta(r - r_0)\right]e^{-i\omega t}\right\}, \\ \mathbf{H}(\mathbf{r},t) &= \mathrm{Re}\left\{\left[\mathbf{H}_{<}(\mathbf{r})\theta(r_0 - r) + \mathbf{H}_{>}(\mathbf{r})\theta(r - r_0)\right]e^{-i\omega t}\right\}, \end{aligned} \tag{2.1}$$

where $\theta(x)$ is the Heaviside step function, $\mathbf{E}_{<}(\mathbf{r})$ $\left[\mathbf{H}_{<}(\mathbf{r})\right]$ and $\mathbf{E}_{>}(\mathbf{r})$ $\left[\mathbf{H}_{>}(\mathbf{r})\right]$ are the electric [magnetic] fields internal and external to the sphere, respectively.

By expanding the electromagnetic field in vectorial spherical harmonics one gets

$$\begin{cases} \mathbf{E}_{<} = \mathbf{E}_{\text{dip}} - \dfrac{ic}{\omega\sqrt{\varepsilon_{\text{in}}}} \displaystyle\sum_{l=0}^{\infty}\sum_{m=-l}^{l} \sqrt{l(l+1)} \left\{ \nabla \times \left[\left[\alpha_{l,m}^{(2,<)} h_l^{(2)}\left(\frac{\omega}{c} r \sqrt{\varepsilon_{\text{in}}}\right)\right] \mathbf{X}_{l,m} \right] \right\}, \\ \mathbf{H}_{<} = \mathbf{H}_{\text{dip}} - \varepsilon_0 c \sqrt{\varepsilon_{\text{in}}} \displaystyle\sum_{l=0}^{\infty}\sum_{m=-l}^{l} \sqrt{l(l+1)} \left\{ \left[\alpha_{l,m}^{(2,<)} h_l^{(2)}\left(\frac{\omega}{c} r \sqrt{\varepsilon_{\text{in}}}\right)\right] \mathbf{X}_{l,m} \right\}, \end{cases}$$

$$\begin{cases} \mathbf{E}_{>} = -\dfrac{ic}{\omega\sqrt{\varepsilon_{\text{out}}}} \displaystyle\sum_{l=0}^{\infty}\sum_{m=-l}^{l} \sqrt{l(l+1)} \left\{ \nabla \times \left[\left[\alpha_{l,m}^{(1,>)} h_l^{(1)}\left(\frac{\omega}{c} r \sqrt{\varepsilon_{\text{out}}}\right)\right] \mathbf{X}_{l,m} \right] \right\}, \\ \mathbf{H}_{>} = -\varepsilon_0 c \sqrt{\varepsilon_{\text{out}}} \displaystyle\sum_{l=0}^{\infty}\sum_{m=-l}^{l} \sqrt{l(l+1)} \left[\alpha_{l,m}^{(1,>)} h_l^{(1)}\left(\frac{\omega}{c} r \sqrt{\varepsilon_{\text{out}}}\right)\right] \mathbf{X}_{l,m}, \end{cases} \tag{2.2}$$

where ε_{out} is the relative dielectric permittivity of the medium places outside the sphere and $\left\{\mathbf{E}_{\text{dip}}, \mathbf{H}_{\text{dip}}\right\}$ is the electromagnetic field radiated by the dipole in the absence of the spherical boundary, given by the expression[1,4]

$$\begin{cases} \mathbf{E}_{\text{dip}} = \left(1 - i\dfrac{\omega}{c}\sqrt{\varepsilon_{\text{in}}} r\right) \dfrac{\mathrm{d}_{\text{dip}} \cos\theta}{2\pi\varepsilon_{\text{in}}\varepsilon_0 r^3} e^{i\frac{\omega}{c}\sqrt{\varepsilon_{\text{in}}} r} \hat{e}_r + \left(1 - i\dfrac{\omega r}{c}\sqrt{\varepsilon_{\text{in}}} - \varepsilon_{\text{in}} \dfrac{\omega^2}{c^2} r^2\right) \dfrac{\mathrm{d}_{\text{dip}} \sin\theta e^{i\frac{\omega}{c}\sqrt{\varepsilon_{\text{in}}} r}}{4\pi\varepsilon_0\varepsilon_{\text{in}} r^3} \hat{e}_\theta, \\ \mathbf{H}_{\text{dip}} = -\dfrac{\omega \mathrm{d}_{\text{dip}}}{4\pi r^2} \sin\theta e^{i\frac{\omega}{c}\sqrt{\varepsilon_{\text{in}}} r} \left(i + \sqrt{\varepsilon_{\text{in}}} \dfrac{\omega r}{c}\right) \hat{e}_\phi . \end{cases} \tag{2.3}$$

By imposing the BCs for the continuity of the normal component of the displacement vector, tangential components of the electric field and continuity of the magnetic field we can calculate the coefficients $\alpha_{l,m}^{(2,<)}$ and $\alpha_{l,m}^{(1,<)}$ by solving the algebraic system

$$\begin{cases} \varepsilon_{\text{in}} \mathbf{E}_< (R,\theta,\phi)\cdot \hat{e}_r = \varepsilon_{\text{out}} \mathbf{E}_> (R,\theta,\phi)\cdot \hat{e}_r \ , \\ \mathbf{E}_< (R,\theta,\phi)\cdot \hat{e}_\theta = \mathbf{E}_> (R,\theta,\phi)\cdot \hat{e}_\theta \ , \\ \mathbf{E}_< (R,\theta,\phi)\cdot \hat{e}_\phi = \mathbf{E}_> (R,\theta,\phi)\cdot \hat{e}_\phi \ , \\ \mathbf{H}_< (R,\theta,\phi)\cdot \hat{e}_r = \mathbf{H}_> (R,\theta,\phi)\cdot \hat{e}_r \ , \\ \mathbf{H}_< (R,\theta,\phi)\cdot \hat{e}_\theta = \mathbf{H}_> (R,\theta,\phi)\cdot \hat{e}_\theta \ , \\ \mathbf{H}_< (R,\theta,\phi)\cdot \hat{e}_\phi = \mathbf{H}_> (R,\theta,\phi)\cdot \hat{e}_\phi \ , \end{cases} \tag{2.4}$$

from which we obtain

$$\begin{cases} \alpha_{1,0}^{(1,>)} = \dfrac{\left\{ \dfrac{1}{\varepsilon_{\text{in}}}\left(1 - i\dfrac{\omega R}{c}\sqrt{\varepsilon_{\text{in}}} - \varepsilon_{\text{in}}\dfrac{\omega^2}{c^2}R^2\right) + \dfrac{\omega^2 R^2}{c^2}\left(1 - \dfrac{ic}{\omega R\sqrt{\varepsilon_{\text{in}}}} - \dfrac{c^2}{\omega^2 R^2 \varepsilon_{\text{in}}}\right)\dfrac{\left(1 - i\dfrac{\omega}{c}\sqrt{\varepsilon_{\text{in}}}R\right)}{\left(1 + i\dfrac{\omega}{c}\sqrt{\varepsilon_{\text{in}}}R\right)} \right\}}{\left\{ \dfrac{1}{\sqrt{\varepsilon_{\text{out}}}}\left(1 + \dfrac{ic}{\omega R\sqrt{\varepsilon_{\text{out}}}} - \dfrac{c^2}{\omega^2 R^2 \varepsilon_{\text{out}}}\right) + \dfrac{1}{\sqrt{\varepsilon_{\text{in}}}}\dfrac{\left(1 + \dfrac{ic}{\omega R\sqrt{\varepsilon_{\text{out}}}}\right)}{\left(1 - \dfrac{ic}{\omega R\sqrt{\varepsilon_{\text{in}}}}\right)}\left(1 - \dfrac{ic}{\omega R\sqrt{\varepsilon_{\text{in}}}} - \dfrac{c^2}{\omega^2 R^2 \varepsilon_{\text{in}}}\right) \right\}} \dfrac{\omega \mathrm{d}_{\text{dip}}}{i\sqrt{12\pi}\varepsilon_0 c R^2} e^{i\frac{\omega}{c}\left(\sqrt{\varepsilon_{\text{in}}} - \sqrt{\varepsilon_{\text{out}}}\right)R} , \\ \alpha_{1,0}^{(2,<)} = \dfrac{\left(1 + \dfrac{ic}{\omega R\sqrt{\varepsilon_{\text{out}}}}\right)}{\left(1 - \dfrac{ic}{\omega R\sqrt{\varepsilon_{\text{in}}}}\right)} e^{i\frac{\omega}{c}R\left(\sqrt{\varepsilon_{\text{in}}} + \sqrt{\varepsilon_{\text{out}}}\right)} \alpha_{1,0}^{(1,>)} + \dfrac{\mathrm{d}_{\text{dip}}\omega^2}{\sqrt{12\pi}\varepsilon_0 R c^2} \dfrac{\left(1 - \dfrac{i\omega R}{c}\sqrt{\varepsilon_{\text{in}}}\right)}{\left(1 - \dfrac{ic}{\omega R\sqrt{\varepsilon_{\text{in}}}}\right)} e^{2i\frac{\omega}{c}\sqrt{\varepsilon_{\text{in}}}R} . \end{cases} \tag{2.5}$$

We are only interested in $\alpha_{1,0}^{(2,<)}$ because it is the amplitude of the far field radiated field by the system, which we will use to calculate the radiative efficiency, see next section.

III. Nanostructure radiative efficiency

In this section we show how to obtain the radiative properties of the system and how to derive the nanostructure radiative efficiency, which is the ratio of the radiated power by the dipole inside a sphere to the one radiated by a dipole in air.

First, we remind that the time averaged Poynting vector in the outer region is given by

$$\mathbf{S}(\mathbf{r},t) = \frac{1}{2}\mathrm{Re}\left\{\mathbf{E}_>(\mathbf{r})\times\mathbf{H}_>^*(\mathbf{r})\right\} = \frac{3\varepsilon_0 c^2}{8\pi\omega r}\left|\alpha_{1,0}^{(1,>)}\right|^2 \sin\theta\,\mathrm{Re}\left\{ i\left(\frac{c}{\omega r\sqrt{\varepsilon_{\text{out}}}} - \frac{ic^2}{\omega^2 r^2 \varepsilon_{\text{out}}}\right) \left\{ \begin{array}{l} -2\cos\theta\left[\dfrac{c}{\omega r\sqrt{\varepsilon_{\text{out}}}} + i\dfrac{c^2}{\omega^2 r^2 \varepsilon_{\text{out}}}\right]\hat{e}_\theta + \\ +\sin\theta\left[-i + \dfrac{c}{\omega r\sqrt{\varepsilon_{\text{out}}}} + i\dfrac{c^2}{\omega^2 r^2 \varepsilon_{\text{out}}}\right]\hat{e}_r \end{array} \right\} \right\} . \tag{3.1}$$

For an electric dipole field in a uniform dielectric, we obtain

$$\mathbf{S}_{\text{dip}}(\mathbf{r},t)=\frac{1}{2}\text{Re}\left\{\mathbf{E}_{\text{dip}}(\mathbf{r})\times\mathbf{H}_{\text{dip}}^{*}(\mathbf{r})\right\}=\frac{\mathrm{d}^{2}{}_{\text{dip}}\omega}{16\pi^{2}\varepsilon_{\text{out}}\varepsilon_{0}r^{5}}\sin\theta\,\text{Re}\left\{\begin{array}{l}\cos\theta\left(1-i\frac{\omega r}{c}\sqrt{\varepsilon_{\text{out}}}\right)\left(-i+\sqrt{\varepsilon_{\text{out}}}\frac{\omega r}{c}\right)\hat{e}_{\theta}+\\+\frac{\sin\theta}{2}\left(i-\sqrt{\varepsilon_{\text{out}}}\frac{\omega r}{c}\right)\left(1-i\frac{\omega r}{c}\sqrt{\varepsilon_{\text{out}}}-\varepsilon_{\text{out}}\frac{\omega^{2}}{c^{2}}r^{2}\right)\hat{e}_{r}\end{array}\right\}. \quad (3.2)$$

In turn we can calculate the radiated power in the far-field region as

$$\begin{cases}P_{\text{rad}}=\int_{0}^{2\pi}d\phi\int_{0}^{\pi}d\theta a^{2}\sin\theta\mathbf{S}(\mathbf{r},t)\cdot\hat{e}_{r}=\frac{\varepsilon_{0}c^{3}}{\omega^{2}\sqrt{\varepsilon_{\text{out}}}}\left|\alpha_{1,0}^{(1,>)}\right|^{2},\\P_{\text{dip}}=\int_{0}^{2\pi}d\phi\int_{0}^{\pi}d\theta a^{2}\sin\theta\mathbf{S}_{\text{dip}}(\mathbf{r},t)\cdot\hat{e}_{r}=\frac{\mathrm{d}_{\text{dip}}{}^{2}\omega^{4}\sqrt{\varepsilon_{\text{out}}}}{12\pi\varepsilon_{0}c^{3}}.\end{cases} \quad (3.3)$$

Finally, we can define the structural radiative efficiency as

$$\eta\equiv\frac{P_{\text{rad}}}{P_{\text{dip}}}=\frac{12\pi\varepsilon_{0}{}^{2}c^{6}}{\mathrm{d}_{\text{dip}}{}^{2}\omega^{6}\varepsilon_{\text{out}}}\left|\alpha_{1,0}^{(1,>)}\right|^{2}, \quad (3.4)$$

which can be rewritten as

$$\eta=4\varepsilon_{\text{out}}e^{-2\kappa_{\text{in}}R}\left|\varepsilon_{\text{in}}^{2}/(\eta_{1}+\eta_{2}+\eta_{3})\right|^{2}, \quad (3.5)$$

where

$$
\begin{aligned}
\kappa_{\text{in}} &= (2\pi/\lambda)\,\text{Im}\sqrt{\varepsilon_{\text{in}}}\ , \\
\eta_1 &= -\left(\lambda^2/4\pi^2R^2\right)\left(\sqrt{\varepsilon_{\text{in}}}+\sqrt{\varepsilon_{\text{out}}}\right)\left(\sqrt{\varepsilon_{\text{in}}}-\sqrt{\varepsilon_{\text{out}}}\right)^2\Big/\sqrt{\varepsilon_{\text{in}}\varepsilon_{\text{out}}}\ , \\
\eta_2 &= i\left(\lambda/2\pi R\right)\left(\varepsilon_{\text{in}}-\varepsilon_{\text{out}}\right)/\varepsilon_{\text{in}}\ , \\
\eta_3 &= \sqrt{\varepsilon_{\text{in}}\varepsilon_{\text{out}}}\left(\sqrt{\varepsilon_{\text{in}}}+\sqrt{\varepsilon_{\text{out}}}\right).
\end{aligned}
\tag{3.6}
$$

IV. Growth of a second spherical shell

Finally, if we consider a dielectric shell coating the previous sphere the fields become

$$
\begin{cases}
\mathbf{E}_1=\mathbf{E}_{\text{dip}}+\mathbf{E}_{1,IN}=\left(1-i\dfrac{\omega}{c}\sqrt{\varepsilon_1}r\right)\dfrac{\text{d}_{\text{dip}}\cos\theta}{2\pi\varepsilon_1\varepsilon_0r^3}e^{i\frac{\omega}{c}\sqrt{\varepsilon_1}r}\hat{e}_r+\left(1-i\dfrac{\omega r}{c}\sqrt{\varepsilon_1}-\varepsilon_1\dfrac{\omega^2}{c^2}r^2\right)\dfrac{\text{d}_{\text{dip}}\sin\theta e^{i\frac{\omega}{c}\sqrt{\varepsilon_1}r}}{4\pi\varepsilon_0\varepsilon_1r^3}\hat{e}_\theta-\dfrac{ic}{\omega\sqrt{\varepsilon_1}}\displaystyle\sum_{l=0}^{\infty}\sum_{m=-l}^{l}\sqrt{l(l+1)}\left\{\nabla\times\left[\left[\alpha_{l,m}^{(2)}h_l^{(2)}\left(\dfrac{\omega}{c}r\sqrt{\varepsilon_1}\right)\right]\mathbf{X}_{l,m}\right]\right\}, \\
\mathbf{H}_1=\mathbf{H}_{\text{dip}}+\mathbf{H}_{1,IN}=-\dfrac{\omega\text{d}_{\text{dip}}}{4\pi r^2}\sin\theta e^{i\frac{\omega}{c}\sqrt{\varepsilon_1}r}\left(i+\sqrt{\varepsilon_1}\dfrac{\omega r}{c}\right)\hat{e}_\phi-\varepsilon_0c\sqrt{\varepsilon_1}\displaystyle\sum_{l=0}^{\infty}\sum_{m=-l}^{l}\sqrt{l(l+1)}\left\{\left[\alpha_{l,m}^{(2)}h_l^{(2)}\left(\dfrac{\omega}{c}r\sqrt{\varepsilon_1}\right)\right]\mathbf{X}_{l,m}\right\},
\end{cases}
\tag{4.1}
$$

$$
\begin{cases}
\mathbf{E}_{2,IN}=\displaystyle\sum_{l=0}^{\infty}\sum_{m=-l}^{l}\sqrt{l(l+1)}\left\{-\dfrac{ic}{\omega\sqrt{\varepsilon_2}}\gamma_{l,m}^{(2)}\nabla\times\left[h_l^{(2)}\left(\dfrac{\omega}{c}r\sqrt{\varepsilon_2}\right)\mathbf{X}_{l,m}\right]\right\}, \\
\mathbf{H}_{2,IN}=\displaystyle\sum_{l=0}^{\infty}\sum_{m=-l}^{l}\sqrt{l(l+1)}\left\{-\varepsilon_0c\sqrt{\varepsilon_2}\gamma_{l,m}^{(2)}h_l^{(2)}\left(\dfrac{\omega}{c}r\sqrt{\varepsilon_2}\right)\mathbf{X}_{l,m}\right\}, \\
\mathbf{E}_{2,OUT}=\displaystyle\sum_{l=0}^{\infty}\sum_{m=-l}^{l}\sqrt{l(l+1)}\left\{-\dfrac{ic}{\omega\sqrt{\varepsilon_2}}\beta_{l,m}^{(1)}\nabla\times\left[h_l^{(1)}\left(\dfrac{\omega}{c}r\sqrt{\varepsilon_2}\right)\mathbf{X}_{l,m}\right]\right\}, \\
\mathbf{H}_{2,OUT}=\displaystyle\sum_{l=0}^{\infty}\sum_{m=-l}^{l}\sqrt{l(l+1)}\left\{-\varepsilon_0c\sqrt{\varepsilon_2}\beta_{l,m}^{(1)}h_l^{(1)}\left(\dfrac{\omega}{c}r\sqrt{\varepsilon_2}\right)\mathbf{X}_{l,m}\right\},
\end{cases}
\tag{4.2}
$$

and

$$
\begin{cases}
\mathbf{E}_3=\mathbf{E}_{3,OUT}=-\dfrac{ic}{\omega\sqrt{\varepsilon_3}}\displaystyle\sum_{l=0}^{\infty}\sum_{m=-l}^{l}\sqrt{l(l+1)}\left\{\nabla\times\left[\left[\gamma_{l,m}^{(1)}h_l^{(1)}\left(\dfrac{\omega}{c}r\sqrt{\varepsilon_3}\right)\right]\mathbf{X}_{l,m}\right]\right\}, \\
\mathbf{H}_3=\mathbf{H}_{3,OUT}=-\varepsilon_0c\sqrt{\varepsilon_3}\displaystyle\sum_{l=0}^{\infty}\sum_{m=-l}^{l}\sqrt{l(l+1)}\left[\gamma_{l,m}^{(1)}h_l^{(1)}\left(\dfrac{\omega}{c}r\sqrt{\varepsilon_3}\right)\right]\mathbf{X}_{l,m}\ ,
\end{cases}
\tag{4.3}
$$

where ε_1 is the dielectric constant of the internal sphere, ε_2 is the dielectric constant of the shell and ε_3 is the dielectric constant out (in our case air).

Boundary conditions for the continuity of the normal component of the displacement vector, tangential component of the electric field and continuity of the magnetic field provide

$$\begin{aligned}
&\varepsilon_1 \mathbf{E}_1(R_1,\theta,\phi)\cdot\hat{e}_r = \varepsilon_2 \mathbf{E}_2(R_1,\theta,\phi)\cdot\hat{e}_r\ ,\\
&\mathbf{E}_1(R_1,\theta,\phi)\cdot\hat{e}_\theta = \mathbf{E}_2(R_1,\theta,\phi)\cdot\hat{e}_\theta\ ,\\
&\mathbf{E}_1(R_1,\theta,\phi)\cdot\hat{e}_\phi = \mathbf{E}_2(R_1,\theta,\phi)\cdot\hat{e}_\phi\ ,\\
&\mathbf{H}_1(R_1,\theta,\phi)\cdot\hat{e}_r = \mathbf{H}_2(R_1,\theta,\phi)\cdot\hat{e}_r\ ,\\
&\mathbf{H}_1(R_1,\theta,\phi)\cdot\hat{e}_\theta = \mathbf{H}_2(R_1,\theta,\phi)\cdot\hat{e}_\theta\ ,\\
&\mathbf{H}_1(R_1,\theta,\phi)\cdot\hat{e}_\phi = \mathbf{H}_2(R_1,\theta,\phi)\cdot\hat{e}_\phi\ ,\\
&\\
&\varepsilon_2 \mathbf{E}_2(R_2,\theta,\phi)\cdot\hat{e}_r = \varepsilon_3 \mathbf{E}_3(R_2,\theta,\phi)\cdot\hat{e}_r\ ,\\
&\mathbf{E}_2(R_2,\theta,\phi)\cdot\hat{e}_\theta = \mathbf{E}_3(R_2,\theta,\phi)\cdot\hat{e}_\theta\ ,\\
&\mathbf{E}_2(R_2,\theta,\phi)\cdot\hat{e}_\phi = \mathbf{E}_3(R_2,\theta,\phi)\cdot\hat{e}_\phi\ ,\\
&\mathbf{H}_2(R_2,\theta,\phi)\cdot\hat{e}_r = \mathbf{H}_3(R_2,\theta,\phi)\cdot\hat{e}_r\ ,\\
&\mathbf{H}_2(R_2,\theta,\phi)\cdot\hat{e}_\theta = \mathbf{H}_3(R_2,\theta,\phi)\cdot\hat{e}_\theta\ ,\\
&\mathbf{H}_2(R_2,\theta,\phi)\cdot\hat{e}_\phi = \mathbf{H}_3(R_2,\theta,\phi)\cdot\hat{e}_\phi\ ,
\end{aligned} \tag{4.4}$$

Thus, we get

$$\begin{bmatrix} \mathrm{A}(\varepsilon_1,R_1) & \mathrm{A}(\varepsilon_2,-R_1) & -\mathrm{A}(\varepsilon_2,R_1) & 0 \\ \mathrm{B}(\varepsilon_1,R_1) & -\mathrm{B}(\varepsilon_2,-R_1) & -\mathrm{B}(\varepsilon_2,R_1) & 0 \\ 0 & -\mathrm{B}(\varepsilon_2,-R_2) & \mathrm{B}(\varepsilon_2,R_2) & \mathrm{B}(\varepsilon_3,-R_2) \\ 0 & -\mathrm{A}(\varepsilon_2,-R_2) & \mathrm{A}(\varepsilon_2,R_2) & \mathrm{A}(\varepsilon_3,-R_2) \end{bmatrix} \begin{bmatrix} \alpha_{1,0}^{(2)} \\ \beta_{1,0}^{(1)} \\ \beta_{1,0}^{(2)} \\ \gamma_{1,0}^{(1)} \end{bmatrix} = \begin{bmatrix} \dfrac{\mathrm{d_{dip}}\omega^3}{3\sqrt{3\pi}\varepsilon_0\varepsilon_1 c^3}\left(\varepsilon_1\dfrac{\omega^2 R_1^2}{c^2} + i\dfrac{\omega R_1}{c}\sqrt{\varepsilon_1} - 1\right) e^{i\frac{\omega}{c}\sqrt{\varepsilon_1}R_1} \\ i\dfrac{\mathrm{d_{dip}}\omega^3}{3\sqrt{3\pi}\varepsilon_0 c^3}\left(i + \dfrac{\omega R_1}{c}\sqrt{\varepsilon_1}\right) e^{i\frac{\omega}{c}\sqrt{\varepsilon_1}R_1} \\ 0 \\ 0 \end{bmatrix}, \tag{4.5}$$

where

$$\begin{cases} \mathrm{A}(\varepsilon,R) = \dfrac{1}{\varepsilon}\left(i\dfrac{\omega^2 R^2}{c^2}\sqrt{\varepsilon} + \dfrac{\omega R}{c} - \dfrac{i}{\sqrt{\varepsilon}}\right) e^{-i\frac{\omega}{c}R\sqrt{\varepsilon}}\ , \\ \mathrm{B}(\varepsilon,R) = \left(\dfrac{\omega R}{c} - \dfrac{i}{\sqrt{\varepsilon}}\right) e^{-i\frac{\omega}{c}R\sqrt{\varepsilon}}\ . \end{cases} \tag{4.6}$$

The time-averaged Poynting vector in the outer region is in turn given by

$$\mathbf{S}(\mathbf{r},t) = \frac{1}{2}\mathrm{Re}\{\mathbf{E}_3(\mathbf{r})\times\mathbf{H}_3^*(\mathbf{r})\} = \frac{c^4\varepsilon_0}{\omega^3\varepsilon_3 R}\frac{27}{32\pi}\left|\gamma_{1,0}^{(1)}\right|^2 \sin\theta\,\mathrm{Re}\left\{\left(i + \frac{c}{\omega R\sqrt{\varepsilon_3}}\right)\left\{\begin{aligned} &-\hat{e}_\theta\left[2\cos\theta\frac{1}{R^2}\left(1 + \frac{ic}{\omega R\sqrt{\varepsilon_3}}\right)\right] + \\ &\hat{e}_r\left[\left(-i\frac{\omega}{Rc}\sqrt{\varepsilon_3} + \frac{1}{R^2} + \frac{ic}{\omega R^3\sqrt{\varepsilon_3}}\right)\sin\theta\right] \end{aligned}\right\}\right\}, \tag{4.7}$$

Which for an electric dipole field becomes

$$\mathbf{S}_{\text{dip}}(\mathbf{r},t)=\frac{1}{2}\text{Re}\left\{\mathbf{E}_{\text{dip}}(\mathbf{r})\times\mathbf{H}_{\text{dip}}^{*}(\mathbf{r})\right\}=\frac{\text{d}^{2}{}_{\text{dip}}\omega}{16\pi^{2}\varepsilon_{3}\varepsilon_{0}r^{5}}\sin\theta\,\text{Re}\left\{\begin{bmatrix}\cos\theta\left(1-i\frac{\omega r}{c}\sqrt{\varepsilon_{3}}\right)\left(-i+\sqrt{\varepsilon_{3}}\frac{\omega r}{c}\right)\hat{e}_{\theta}+\\+\frac{\sin\theta}{2}\left(i-\sqrt{\varepsilon_{3}}\frac{\omega r}{c}\right)\left(1-i\frac{\omega r}{c}\sqrt{\varepsilon_{3}}-\varepsilon_{3}\frac{\omega^{2}}{c^{2}}r^{2}\right)\hat{e}_{r}\end{bmatrix}\right\}. \quad (4.8)$$

The radiated power in the far-field region is in turn given by

$$\begin{aligned}P_{\text{rad}}&=\int_{0}^{2\pi}d\phi\int_{0}^{\pi}d\theta a^{2}\sin\theta\mathbf{S}(\mathbf{r},t)\cdot\hat{e}_{r}=\frac{9}{4}\frac{c^{3}\varepsilon_{0}}{\omega^{2}\sqrt{\varepsilon_{3}}}\left|\gamma_{1,0}^{(1)}\right|^{2},\\P_{\text{dip}}&=\int_{0}^{2\pi}d\phi\int_{0}^{\pi}d\theta a^{2}\sin\theta\mathbf{S}_{\text{dip}}(\mathbf{r},t)\cdot\hat{e}_{r}=\frac{\text{d}_{\text{dip}}{}^{2}\omega^{4}\sqrt{\varepsilon_{3}}}{12\pi\varepsilon_{0}c^{3}},\end{aligned} \quad (4.9)$$

The nanostructure radiative efficiency, remembering that $\varepsilon_3=\varepsilon_{\text{out}}$, is in turn given by

$$\eta=\frac{27\pi c^{6}\varepsilon_{0}^{2}\left|\gamma_{1,0}^{(1)}\right|^{2}}{\text{d}_{\text{dip}}{}^{2}\omega^{6}\varepsilon_{\text{out}}}=\frac{27\pi c^{6}\varepsilon_{0}^{2}}{\text{d}_{\text{dip}}{}^{2}\left(2\pi/\lambda\right)^{6}\varepsilon_{\text{out}}}\left|\gamma_{1,0}^{(1)}\right|^{2}. \quad (4.10)$$

Supplementary figures:

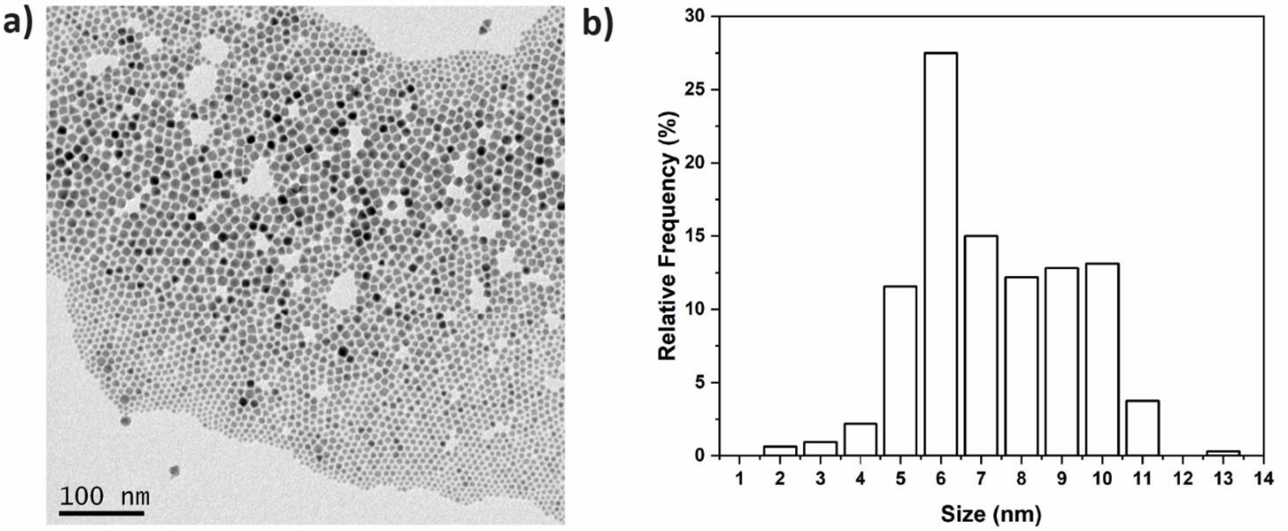

Figure S1: Transmission electron microscopy characterization of the CdSe/CdS sample used as seeds for all the silica growth experiments of this work: a) Representative TEM bright field image of a toluene dispersed sample and b) its diameter length dispersion graph.

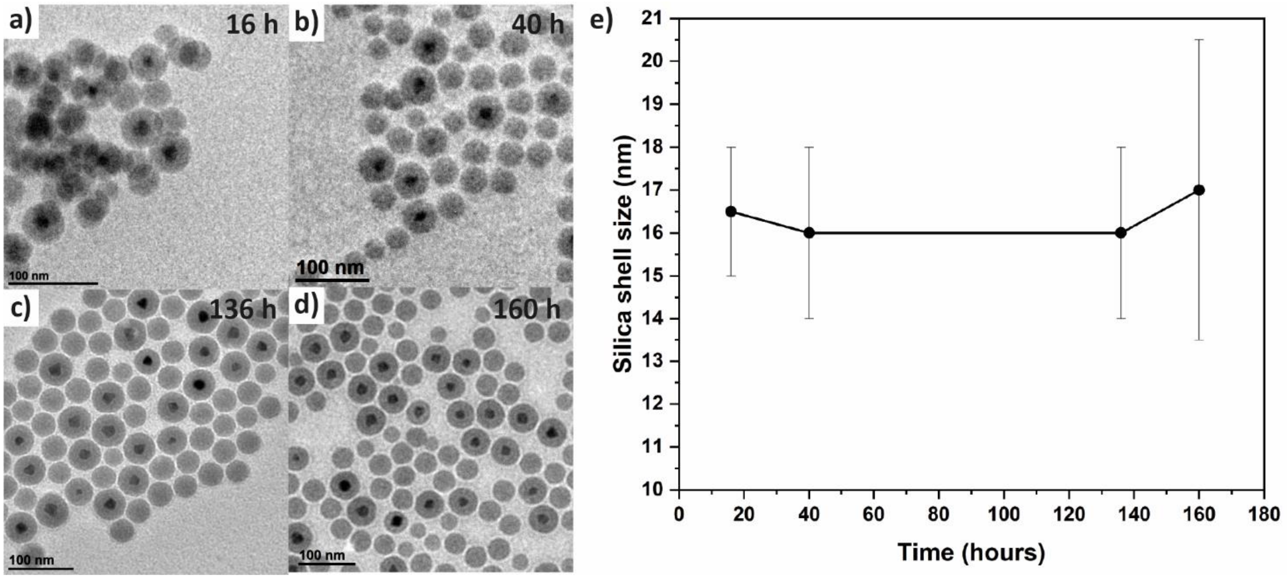

Figure S2: a-d) Representative TEM bright field image of a silica coated CdSe/CdS sample after 16 (a), 40 (b), 136 (c) and 160 (d) hours of silica growth procedure; e) Silica shell size evolution vs time graph.

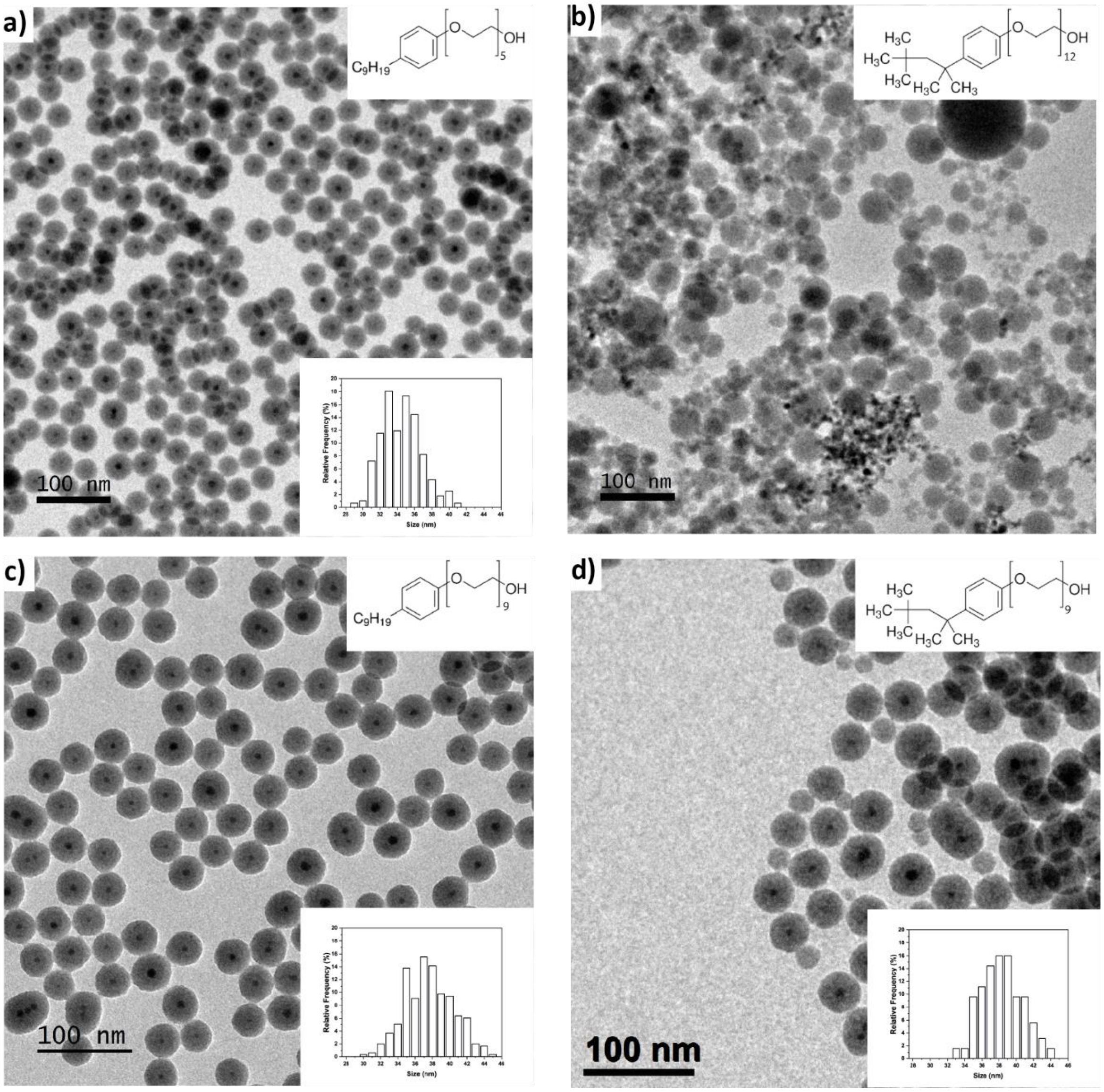

Figure S3: Representative TEM bright field images of silica coated CdSe/CdS samples obtained using different surfactants; upper inserts: chemical formula of the used surfactant, lower insert: diameter length dispersion graph. a) Polyoxyethylene (5) nonylphenylether, b) Polyoxyethylene (9) nonylphenylether, c) Polyoxyethylene (9) isooctylphenylether, d) Polyoxyethylene (12) isooctylphenylether.

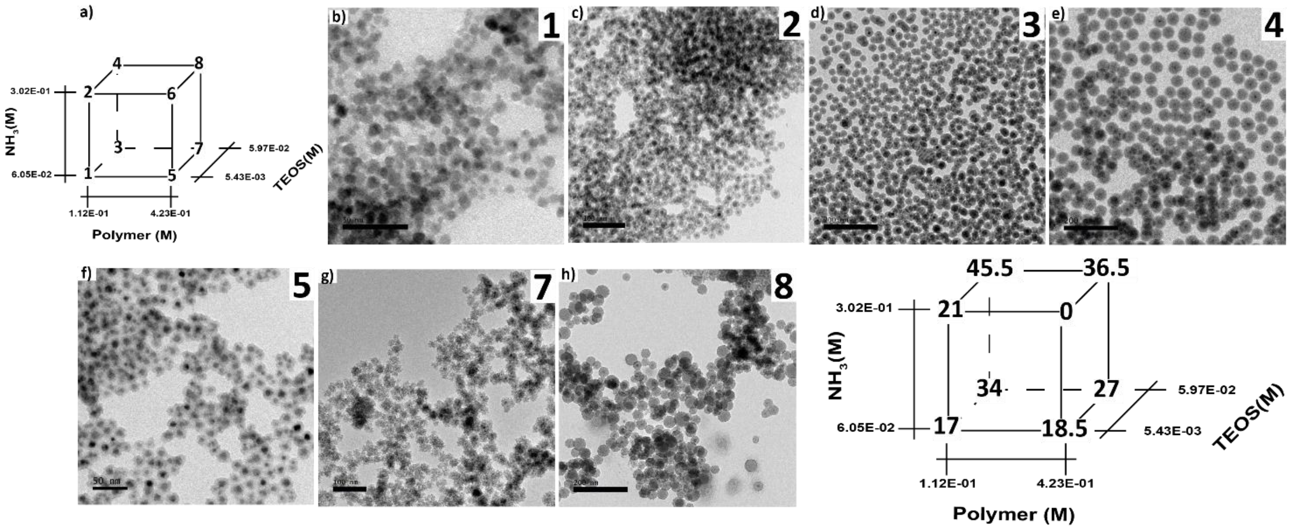

Figure S4: a) schematic of the first set of DOE experiments with indication of samples' number (b-h) representative TEM bright field images of the resulting samples, top-right inserts: sample number b) sample number 1 c) sample number 2 d) sample number 3 e) sample number 4 f) sample number 5 g) sample number 7 h) sample number 8 i) schematic of the first set of DOE experiments with indication of samples' average diameter size.

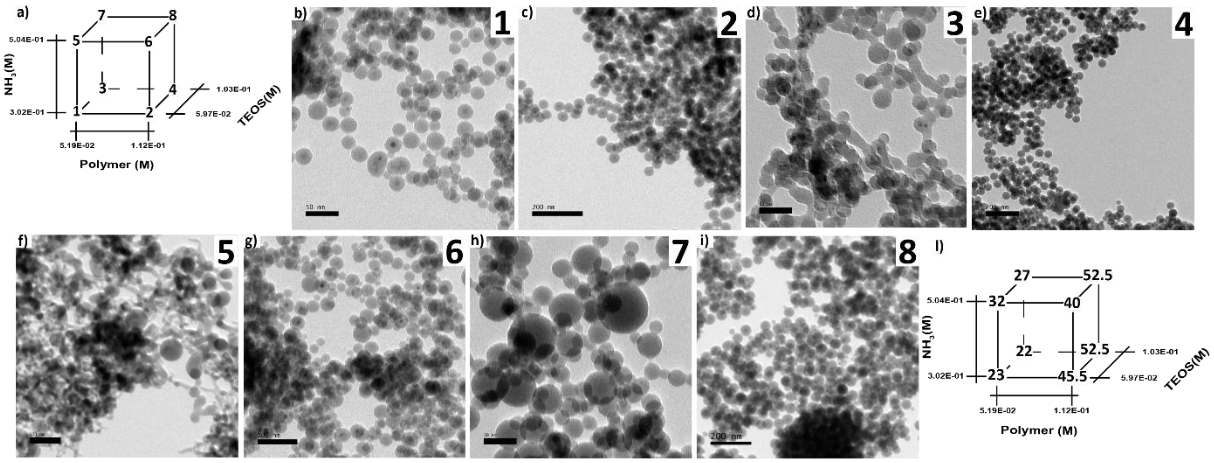

Figure S5: a) schematic of the second set of DOE experiments with indication of samples' number (b-i) representative TEM bright field images of the resulting samples, top-right inserts: sample number b) sample number 1 c) sample number 2 d) sample number 3 e) sample number 4 f) sample number 5 g) sample number 6 h) sample number 7 i) sample number 8 l) schematic of the second set of DOE experiments with indication of samples' average diameter size.

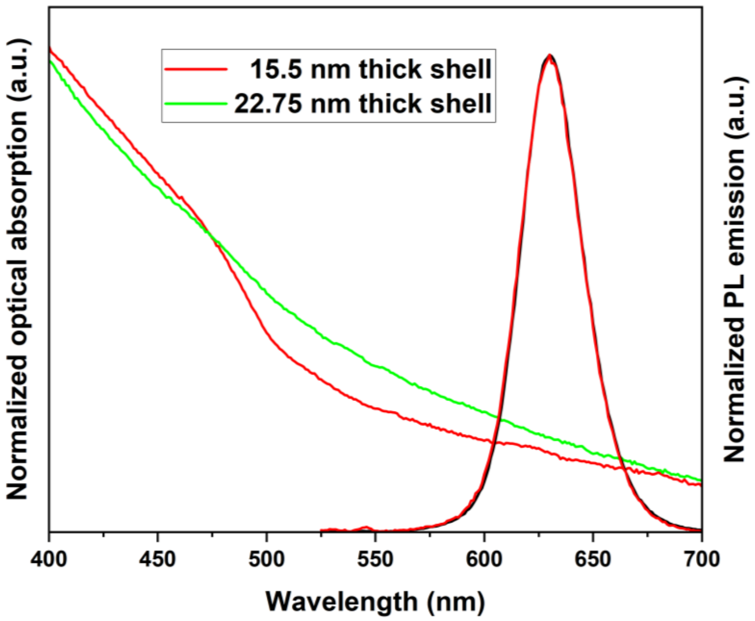

Figure S6: Normalized optical absorption and PL emission of silica coated CdSe/CdS nanocrystals before (black curves) and after (red curves) silica shelling procedure optimization through DOE.

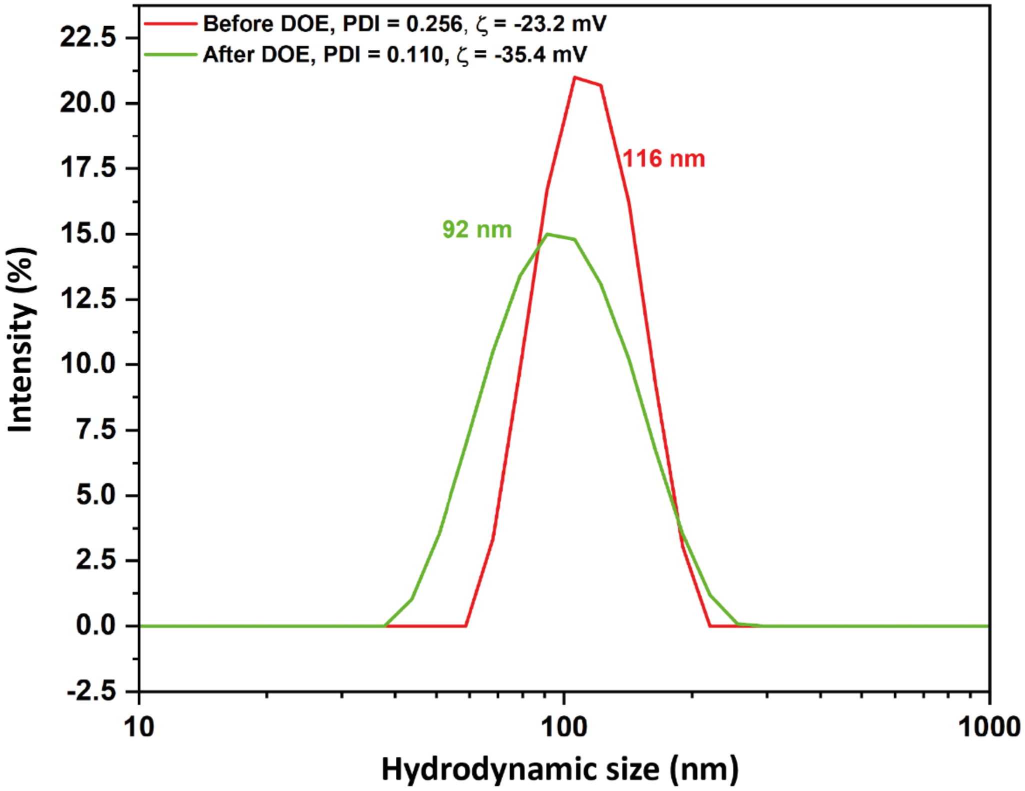

Figure S7: Dynamic light scattering size dispersion graph (by intensity) of the sample obtained before (red line) and after (green line) silica shelling procedure optimization through DOE.

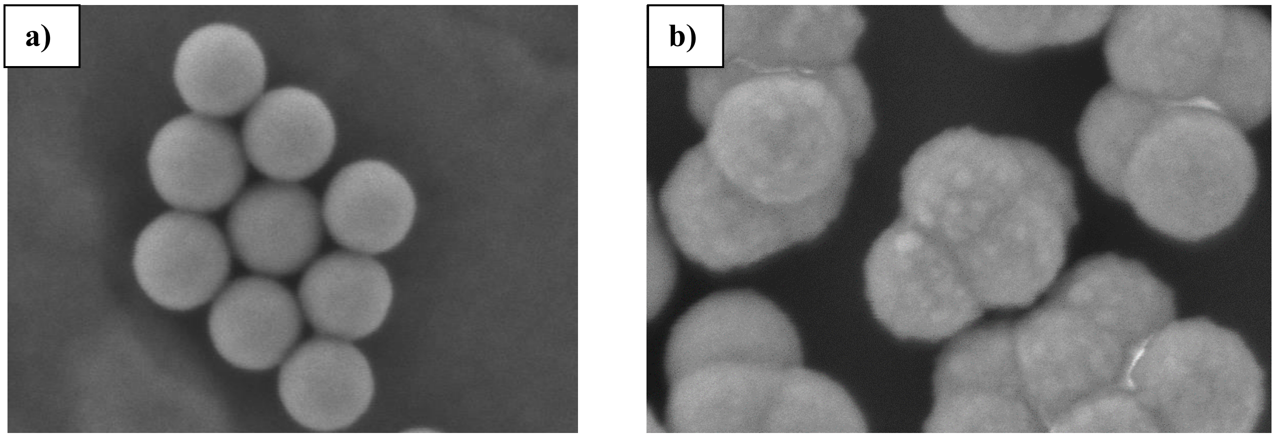

Figure S8: Representative TEM images of the surface of silica coated nanoparticles whose last silica shell was grown through a) reverse microemulsion procedure, b) Stöber procedure.

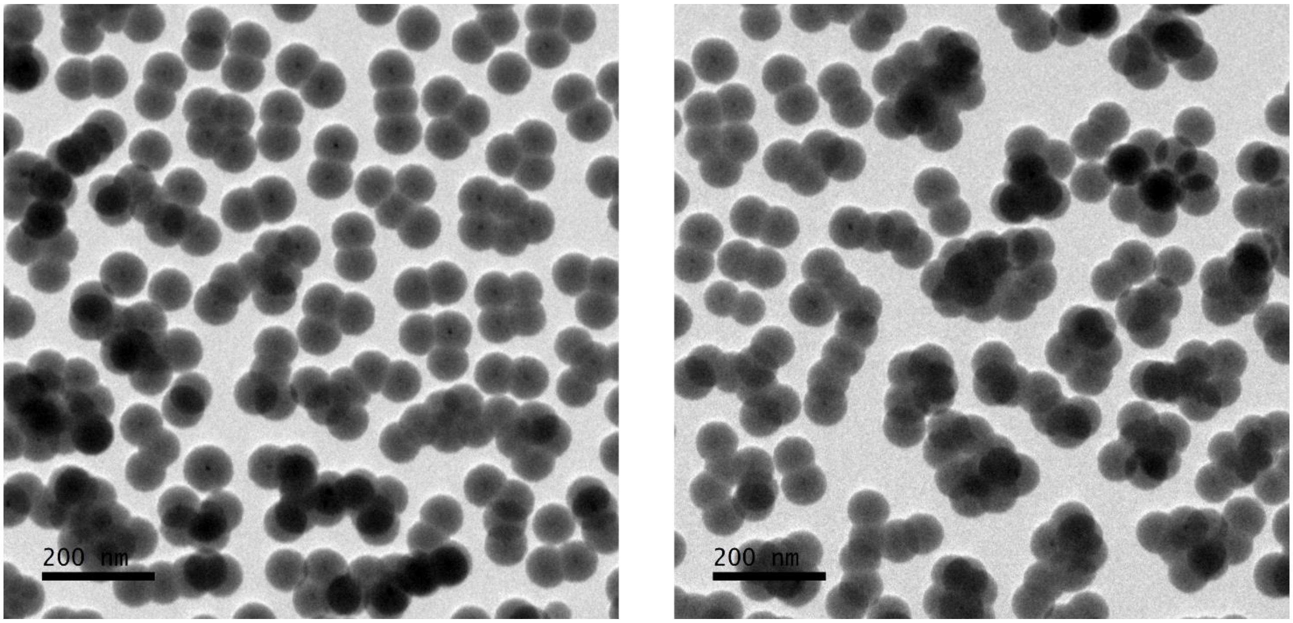

Figure S9: Representative bright field TEM images of silica coated CdSe/CdS nanoparticles obtained by 1 step of reverse microemulsion approach followed by one step of Stöber procedure.

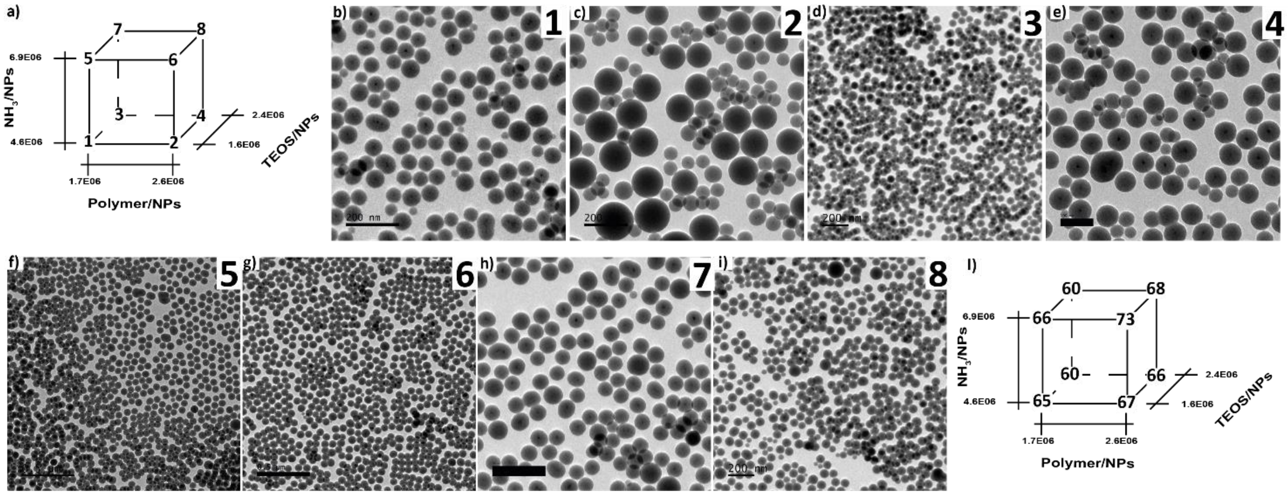

Figure S10: a) schematic of the set of DOE experiments performed for the second injection performed with reverse-microemulsion procedure with indication of samples' number (b-i) representative TEM bright field images of the resulting samples, top-right inserts: sample number b) sample number 1 c) sample number 2 d) sample number 3 e) sample number 4 f) sample number

5 g) sample number 6 h) sample number 7 i) sample number 8 l) schematic of the set of DOE experiments with indication of samples' average diameter size.

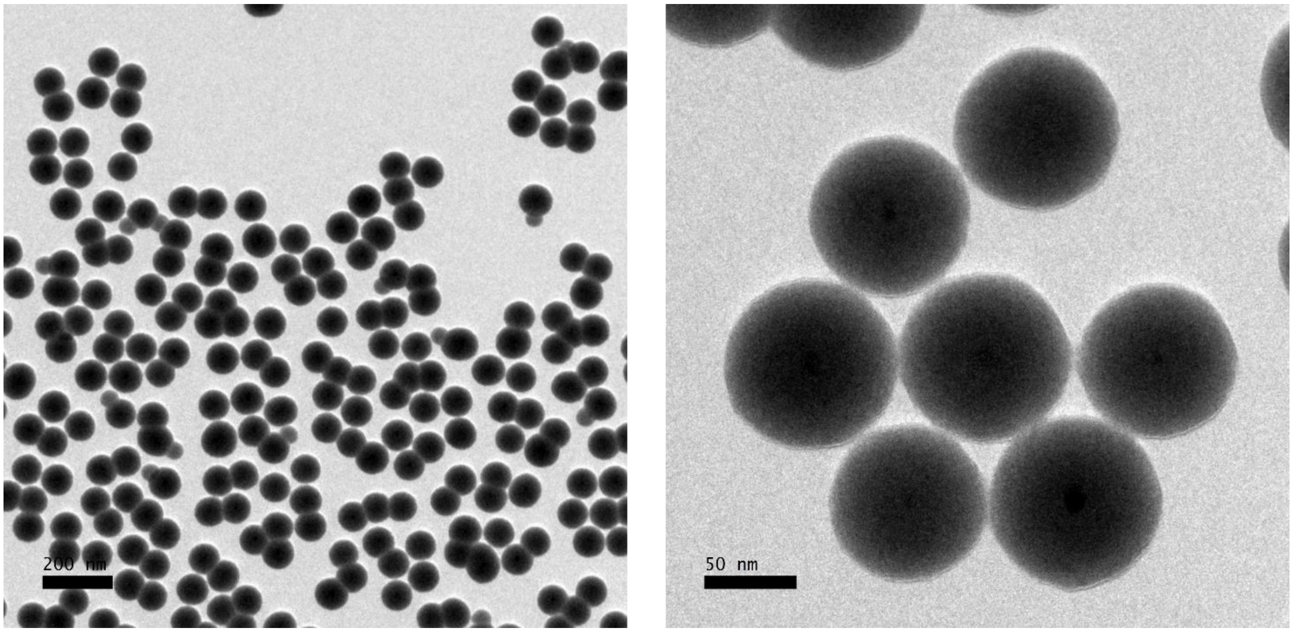

Figure S11: Representative bright field TEM images of silica coated CdSe/CdS nanoparticles obtained by 2 sequential steps of reverse microemulsion approach followed by one step of Stöber procedure.

Table S1: Level and corresponding concentrations of the eight samples of first DOE

**Experiment**	**Polymer**		**TEOS**		**NH_3**	
**Number**	**Level**	**[M]**	**Level**	**[M]**	**Level**	**[M]**
**1**	**-1**	1.12×10^{-1}	**-1**	5.43×10^{-3}	**-1**	6.05×10^{-2}
**2**	**-1**	1.12×10^{-1}	**-1**	5.43×10^{-3}	**+1**	3.02×10^{-1}
**3**	**-1**	1.12×10^{-1}	**+1**	5.97×10^{-2}	**-1**	6.05×10^{-2}
**4**	**-1**	1.12×10^{-1}	**+1**	5.97×10^{-2}	**+1**	3.02×10^{-1}
**5**	**+1**	4.23×10^{-1}	**-1**	5.43×10^{-3}	**-1**	6.05×10^{-2}
**6**	**+1**	4.23×10^{-1}	**-1**	5.43×10^{-3}	**+1**	3.02×10^{-1}
**7**	**+1**	4.23×10^{-1}	**+1**	5.97×10^{-2}	**-1**	6.05×10^{-2}
**8**	**+1**	4.23×10^{-1}	**+1**	5.97×10^{-2}	**+1**	3.02×10^{-1}

Table S2: Level and corresponding concentrations of the eight samples of second DOE

**Experiment**	**Polymer**		**TEOS**		**NH_3**	
**Number**	**Level**	**[M]**	**Level**	**[M]**	**Level**	**[M]**
**1**	**-1**	5.19×10^{-2}	**-1**	5.97×10^{-2}	**-1**	3.02×10^{-1}
**2**	**+1**	1.12×10^{-1}	**-1**	5.97×10^{-2}	**-1**	3.02×10^{-1}
**3**	**-1**	5.19×10^{-2}	**+1**	1.03×10^{-1}	**-1**	3.02×10^{-1}
**4**	**+1**	1.12×10^{-1}	**+1**	1.03×10^{-1}	**-1**	3.02×10^{-1}
**5**	**-1**	5.19×10^{-2}	**-1**	5.97×10^{-2}	**+1**	5.04×10^{-1}
**6**	**+1**	1.12×10^{-1}	**-1**	5.97×10^{-2}	**+1**	5.04×10^{-1}
**7**	**-1**	5.19×10^{-2}	**+1**	1.03×10^{-1}	**+1**	5.04×10^{-1}
**8**	**+1**	1.12×10^{-1}	**+1**	1.03×10^{-1}	**+1**	5.04×10^{-1}

Table S3: Level and corresponding ratios with NPs concentration of the eight samples of third

Experiment	**Polymer/NPs**		**TEOS/NPs**		**NH_3/NPs**	
Number	**Level**	**$[M]_P/[M]_{NPs}$**	**Level**	**$[M]_P/[M]_{NPs}$**	**Level**	**$[M]_P/[M]_{NPs}$**
1	**-1**	1.7×10^6	**-1**	1.6×10^6	**-1**	4.6×10^6
2	**+1**	2.6×10^6	**-1**	1.6×10^6	**-1**	4.6×10^6
3	**-1**	1.7×10^6	**+1**	2.4×10^6	**-1**	4.6×10^6
4	**+1**	1.12×10^{-1}	**+1**	2.4×10^6	**-1**	4.6×10^6
5	**-1**	1.7×10^6	**-1**	1.6×10^6	**+1**	6.9×10^6
6	**+1**	1.12×10^{-1}	**-1**	1.6×10^6	**+1**	6.9×10^6
7	**-1**	1.7×10^6	**+1**	2.4×10^6	**+1**	6.9×10^6
8	**+1**	1.12×10^{-1}	**+1**	2.4×10^6	**+1**	6.9×10^6

References:

(1) Jackson, J. D. *Classical Electrodynamics*, 3rd ed.; Wiley: New York, 1999.

(2) Chew, W. C. *Waves and Fields in Inhomogeneous Media*; IEEE Press: New York, 1995.

(3) Gradshteyn, I. S.; Ryzhik, I. M. *Table of Integrals, Series, and Products*, 7th ed.; Academic Press: Cambridge, 2007.

(4) Novotny, L.; Hecht, B. *Principles of Nano-Optics*; Cambridge University Press: Cambridge, 2006.
